# Synthesis, Structure, and Antitumor Activity of Heterocyclic 2-[4-(Dimethylamino)benzyl]-3-oxoisoindoline-4-carboxylates

**DOI:** 10.3390/molecules31091528

**Published:** 2026-05-05

**Authors:** Gulim K. Mukusheva, Roza I. Jalmakhanbetova, Zharkyn Zh. Zhumagaliyeva, Gulzhaukhar A. Toktarbay, Irina A. Kolesnik, Ekaterina A. Akishina, Evgenij A. Dikusar, Vladimir I. Potkin, Aliaksandr L. Pushkarchuk, Tatiana I. Terpinskaya, Fedor I. Zubkov, Mikhail S. Grigoriev, Hongwei Zhou

**Affiliations:** 1 Chemistry Faculty, NLC “Karaganda National Research University Named After Academician Ye.A. Buketov”, Karaganda 100024, Kazakhstan; mukushevagulim5@gmail.com (G.K.M.); gulzhaukhar2001@gmail.com (G.A.T.); 2 Department of Chemistry, Institute of Natural Sciences, L.N. Gumilyov Eurasian National University, Astana 010000, Kazakhstan; rjalmakhanbetova@gmail.com; 3 Astana International University, Astana 010000, Kazakhstan; zharkyn.73@mail.ru; 4 Laboratory of Chemistry of Heterocyclic Compounds, Institute of Physical Organic Chemistry, National Academy of Sciences of Belarus, 220072 Minsk, Belarus; che.semenovaea@mail.ru (E.A.A.); evgen_58@mail.ru (E.A.D.); potkin@ifoch.bas-net.by (V.I.P.); 5 Laboratory of Ion Exchange and Sorption, Institute of Physical Organic Chemistry, National Academy of Sciences of Belarus, 220072 Minsk, Belarus; alexp51@bk.ru; 6 Center of Medical Microbiology and Antibiotic Resistance, Institute of Physiology, National Academy of Sciences of Belarus, 220072 Minsk, Belarus; terpinskayat@mail.ru; 7 Faculty of Science, Peoples Friendship University of Russia (RUDN University), Moscow 117198, Russia; fzubkov1973@gmail.com; 8 Frumkin Institute of Physical Chemistry and Electrochemistry, Russian Academy of Sciences, Moscow 119071, Russia; mickgrig@mail.ru; 9 College of Chemistry, Biology and Chemical Engineering, Jiaxing University, Jiaxing 314001, China; zhouhw@zju.edu.cn

**Keywords:** isoindoline, isoxazole, isothiazole, esters, quaternization, drug synergism, quantum chemical modeling

## Abstract

In this study, the synthesis of a series of alkaloid analogs—isoxazole and isothiazole esters of 2-substituted 3-oxoindoline-4-carboxylic acid was performed. The target derivatives were obtained by the carbodiimide method. It was established that the studied esters have low cytotoxicity and are able to enhance the effect of the anticancer drug carboplatin, taken in low doses (0.5–5 μM), by up to 30%. Quantum chemical modeling of the obtained compounds and their conjugates with carboplatin was carried out to analyze the relationship between various calculated parameters and the observed biological effects.

## 1. Introduction

Isoxazole and isothiazole heterocycles occupy a special place among pharmacophore fragments and functional groups. Their natural and synthetic derivatives have a very broad spectrum of biological activity, including antibacterial, anti-inflammatory, immunomodulating, antioxidant, antiviral, antitumor, and other properties [1,2]. These heterocycles are a structural fragment of such therapeutics as the antibiotics sulfamethoxazole, sulfisoxazole, and oxacillin [3]; anti-inflammatory drugs such as valdecoxib [1] and leflunomide [4]; antiseizure medication such as zonisamide [5]; and antipsychotics such as paliperidone [6], ziprasidone [7], and perospirone [8].

**Figure 1:**
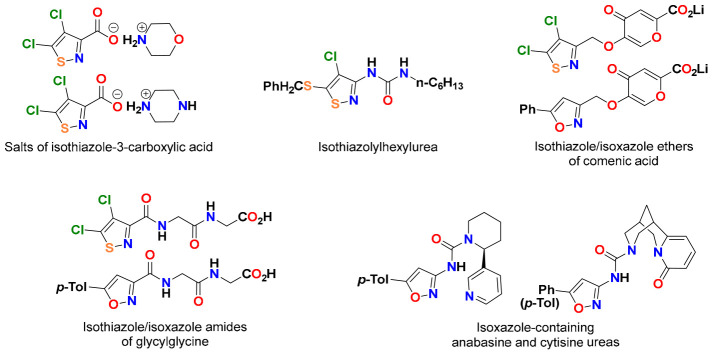
Structures of azole derivatives that exhibited a synergistic effect in combination with antitumor drugs used in cancer therapy.

One more important and unique feature of isoxazole and isothiazole derivatives is their ability to enhance the action of first-line antitumor drugs. Thus, synergism was observed for the morpholine/piperazine salts of 4,5-dichloroisothiazole-3-carboxylic acid and cisplatin [9,10]; for isothiazolylhexylurea with cisplatin, cytarabine, and etoposide [10]; for isoxazole/isothiazole ethers of comenic acid and temozolomide [11]; for isoxazole/isothiazole amides of glycylglycine and doxorubicin [12]; and for isoxazole-containing anabasine and cytisine ureas with ribomustine [13] (Figure 1). The strategy of reducing the dosage of toxic active substances while maintaining a drug’s effectiveness through the use of synergists may help improve the efficiency of antitumor therapy and the quality of the patient’s life, so this is a promising area of research. However, it is characteristic that the presence or absence of a synergistic effect in azole derivatives is currently difficult to predict. It strongly depends on the specific type of tumor cells and the therapeutic agents, as well as the structure of the synergist used. In order to put this phenomenon to the service of medicine, it is necessary to explain the mechanism of the effect, which, in turn, is impossible without the accumulation of a sufficient array of experimental data.

As noted above, a synergistic effect has been repeatedly observed with azole derivatives of alkaloids. This makes substances of this type promising objects for accumulating the required data. The core of many natural alkaloids and their synthetic analogs with medicinal properties is the oxidized or reduced isoindole scaffold [14]. The aim of our study was to synthesize new heterocyclic derivatives of the isoindole series and investigate their antitumor activity, including the presence of a synergistic effect when used in combination with known antitumor drugs.

Here we report the ability to enhance the effect of first-line antitumor drugs found in isoxazole and isothiazole esters of 2-[4-(dimethylamino)benzyl]-3-oxoisoindoline-4-carboxylic acid, which were synthesized, described, and biotested for the first time. Based on a comparison of the observed biological effects with data from quantum chemical calculations, assumptions are made regarding the possible mechanism of the phenomenon.

## 2. Results and Discussion

### 2.1. Synthesis

**Scheme 1:**
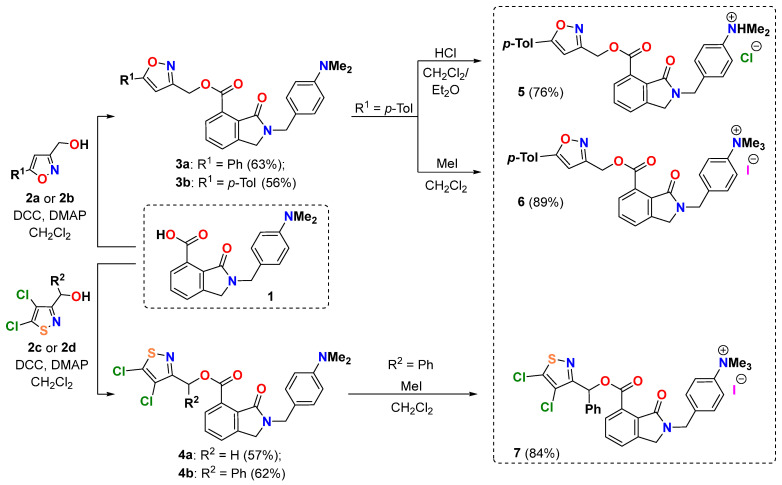
Synthesis of azole-containing 3-oxoisoindoline-4-carboxylic acid esters and their quaternization.

The Steglich esterification procedure was used to obtain the target esters of 2-substituted 3-oxoisoindoline-4-carboxylic acid **1** (Scheme 1). Namely, the starting acid **1** was reacted with 5-aryl-3-hydroxymethylisoxazoles (**2a** or **2b** [15]) or 4,5-dichloro-3-hydroxymethylisothiazoles (**2c** or **2d** [16]) in dichloromethane in the presence of N,N′-dicyclohexylcarbodiimide (DCC) and 4-dimethylaminopyridine (DMAP). The applicability of this method to the reactions involving similar epoxyisoindolinecarboxylic acids was shown by us earlier [17]. The carbodiimide approach was chosen because we failed to obtain 3-oxoisoindoline-4-carboxylic acid chloride with common methods. The use of thionyl chloride as a chlorinating agent was accompanied by resinification of the reaction mixture, whereas when oxalyl chloride was used, the hydrochloride of the starting acid precipitated from the reaction mixture. In turn, the Steglich method [18] allowed the acid to be introduced into the reaction without further modification. Moreover, the starting acid **1** and the reaction products were very soluble in dichloromethane, one of the popular solvents for this type of reaction, whereas dicyclohexylurea is insoluble and facilitated the separation of the reaction products. However, the method used was not without its drawbacks. The side processes that are characteristic of the synthesis of esters by the carbodiimide method proceeded in parallel, so that the yield of the target products **3** and **4** was moderate (56–63%).

Hydrochloride **5** was prepared by passing hydrogen chloride through a solution of the ester **3b** in the mixture of dichloromethane and diethyl ether. Methyl iodide salts **5** and **6** were obtained by prolonged treatment of the starting esters **3b** and **4b** with iodomethane in methylene chloride, followed by the removal of the solvent and washing of the residue with diethyl ether.

The formation of the target compounds was confirmed by comparison of the IR spectra of the starting materials and the products, as well as by NMR analysis. In the IR spectra of the esters **3**–**4**, all substances have two intense bands of C-O-C bonds stretching vibrations at 1279–1290 cm^−1^ and 1133–1136 cm^−1^ in their spectra, which were absent in the spectrum of the starting acid **1**. In the ^1^H NMR spectra of esters **3** and **4**, introduction of the azole fragment was evidenced by the signals of the azole-linked methylene group in **3a**, **3b**, and **4a** (5.56–5.57 ppm), a tertiary proton in the derivative **4b** (7.23 ppm), and protons of the isoxazole rings in **3a** and **3b** (6.92–6.99 ppm). In the ^13^C NMR spectra, carbons from azole-linked methylen groups in **3a**, **3b**, and **4a** were manifested at 59.1–62.3 ppm, from the isoxazole CH group in **3a** and **3b**, at 99.5–100.1 ppm, while the signal from the tertiary carbon adjacent to isothiazole in **4b** was observed at 74.4 ppm.

The quaternization of esters was accompanied by noticeable changes in the spectra of the target compounds, with the most significant changes in the position of signals related to the isoindoline part of the molecules. In the ^1^H NMR spectra of salts **5**–**7**, a general downfield shift in proton signals was observed. The methyl groups of the quaternized dimethylamine substituent were shifted by 0.7 ppm for methyl iodides **6** and **7**, and 0.2 ppm for hydrochloride **5**. The signals of the methylene groups of the isoindoline fragment were found at 4.47–4.50 ppm and 4.76–4.81 ppm instead of 4.19–4.21 ppm and 4.65–4.71 ppm for the starting esters. In the ^13^C NMR spectra, iodomethylation resulted in a considerable downfield shift in the signal from the carbon of methyl groups (appeared at 57.0 ppm both for **6** and **7** instead of 40.7 ppm). A similar signal in the spectrum of the hydrochloride **5** did not shift as much and was observed at 46.8 ppm (instead of 46.1 ppm for **3b**).

**Figure 2:**
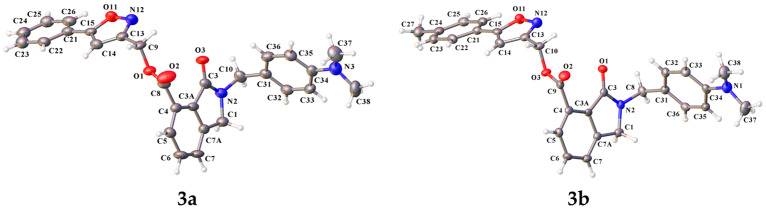
The molecular structure of isoxazole 2-[4-(dimethylamino)benzyl]-3-oxoisoindoline-4-carboxylates (**3a** and **3b**) with atoms represented as thermal vibration ellipsoids of 50% probability.

The formation of esters **3** and **4**, as well as quaternized derivatives **5**–**7**, was also confirmed by mass spectrometry and elemental analysis. Moreover, the exact spatial structure of esters **3a** and **3b** has been established by X-ray data. Crystal data and details of their structure refinement are given in Tables S1–S7; crystal packing diagrams are provided in Figure S1 (see Supplementary Materials), whereas the structures themselves are shown in Figure 2.

### 2.2. Cytotoxicity Evaluation

The cytotoxic activity of substances **3**–**7** was studied in vitro on the larvae of the marine crustacean *Artemia salina*. This type of testing is used to assess the potential of the general cytotoxic effect of various compounds [19,20]. The results obtained are presented in Table 1.

As can be seen from the comparison of LD_50_ values, the cytotoxic effect of esters **3** and **4** appeared to be 5.4–6 times lower than that of dactinomycin. Thus, isoxazole and isothiazole esters of N-substituted 3-oxoisoindoline-4-carboxylic acid **1** are very low in cytotoxicity by themselves. In turn, hydrochloride **5** showed only 3.8 times less activity compared to dactinomycin and was 1.4 times more active than the initial ester **3b**. Methyl iodides **6** and **7** (acted 3.1–3.3 times weaker than dactinomycin) were a bit less cytotoxic than hydrochloride **5** (1.1–1.2 times) and were more cytotoxic than the starting esters **3b** and **4b** (1.6 and 1.8 times, respectively). Most likely, the difference in the cytotoxic effect of the esters **3b** and **4b** and their quaternized forms is due to the difference in solubility. This confirms the initial hypothesis that such a method of additional modification as quaternization will increase the polarity and bioavailability, and therefore the bioactivity, of the compounds obtained.

### 2.3. Evaluation of Antitumor Activity

The anticancer activity of derivatives **5**–**7**, known antitumor drugs [carboplatin (Cb), doxorubicin (Dx), cyclophosphamide (Cp), fluorouracil (Fu)], and their binary mixtures was determined on human cervical cancer (HeLa) and rat glioma cell lines using the standard MTT assay procedure [21]. We have previously described the synergistic effect of isothiazole and isoxazole derivatives with cisplatin [9,10]. Carboplatin has a structure similar to cisplatin and has a similar mechanism of action, but is less toxic. The other chemotherapeutic agents listed above were chosen to assess the presence of synergism between specific azole derivatives and drugs that act through other pathways.

Firstly, the intrinsic antitumor effect of the synthesized salts **5**–**7**, taken at a concentration of 200 µM, and their action in combination with carboplatin were investigated. To obtain a better understanding of the influence of the concentration of the chemotherapeutic agent and the compounds under study on the observed effect, the experiments were also conducted using compounds **5**–**7** at 2-times lower concentration (100 µM) and carboplatin at a concentration of 40 µM. The initial data on tumor cell survival are presented in Figures S2–S4 (see Supplementary Materials). The obtained values were converted into a suppression index according to the following formula: decrease in viable cell concentration (%) = 100% − cell viability (%). The verdict on the observed effect was based on the difference between the combined and sum effects of carboplatin and the test compound: if the combined suppression is greater than the sum one, synergism is observed; if less, antagonism. The results obtained are presented in Table 2 and Table 3.

As can be seen from Table 2 and Table 3, compounds **5**–**7** at a concentration of 100 and 200 µM suppressed the growth of glioma C6 and HeLa. Glioma C6 cells were more sensitive to compounds **5** and **7**, while HeLa cells were more sensitive to compounds **6** and **7**. In the case of compound **5**, its own effect on glioma C6 cells is dose-dependent and is higher at a concentration of 200 μM than at 100 μM, whereas on HeLa cells, as well as in the case of compounds **6** and **7** for both cell lines, the effect is approximately the same at both concentrations. However, taking the fairly high doses and relatively low decrease in cell viability into account, it can be considered that the antiproliferative effect of compounds **5**–**7** on their own is rather weak.

At a concentration of 200 μM, compound **5** was found to be most active against glioma C6 cells and least active against HeLa cells: the suppression of cell viability was about 38% and 20%, respectively. In the case of glioma C6 cells, the effect of using carboplatin at a dose of 5 μM in combination with compound **5** (200 µM) was 10% higher than the sum of their effects, whereas in the case of HeLa cells, it was 23% higher. The observed enhancement suggests the manifestation of synergism. The effect of the combined use of derivative **5** (100 µM or 200 µM) and carboplatin at the concentrations of 0.5 µM and 10 µM on both cell lines, as well as at 20 µM and 40 µM on glioma C6 cells, was actually equal to the summarized effect of these compounds on their own. On the contrary, when compound **5** was used in combination with carboplatin taken at concentrations of 20 µM and 40 µM taken for glioma C6, a noticeable increase in action was observed in comparison to the summarized effect (15% and 13%).

Compound **6** on its own, at a concentration of 200 μM, was approximately 1.7 times more active against HeLa cells and about 2 times less active against glioma cells than derivative **5** (34.8% and 22.1% decrease in cell viability, correspondingly). When compound **6** was co-administered with carboplatin at a concentration of 10–40 μM on C6 glioma cells, virtually no effects were observed, since the result of co-administration was close to the sum of the effects of the individual components, whereas weak antagonism was observed on HeLa cells at 10 μM and 20 μM doses of carboplatin (13% and 17%). However, at very low doses of carboplatin, an increase in the combined effect of carboplatin and derivative **6** relative to their summarized effect was observed. At a dose of 0.5 μM carboplatin, the effect was observed only in C6 glioma cells (an increase of 22%), and at a dose of 5 μM, it was observed in both cell lines (an increase of 17% in the case of HeLa and 30% in glioma cells).

Compound **7** at a concentration of 200 μM was the most active against HeLa cells (58.1% decrease in cell viability) and was only slightly inferior to compound **5** in its effect on C6 glioma (34.9% decrease in cell viability against 38.2% in the last case). In compositions with carboplatin, derivative **7** behaved similarly to compound **6**. When carboplatin was taken in concentrations of 10–40 μM, in all cases, the result of its combined use with compound **7** on HeLa cells was somewhat lower than their combined effect (5–20%), indicating the manifestation of antagonism. In the case of glioma cells, the combined effect of carboplatin at 10 μM and 20 μM was almost equal to their combined effect, and even weak antagonism was observed during the co-administration of a 40 μM dose of carboplatin and a 100 μM dose of compound **7**. However, when the combination with carboplatin at a concentration of 0.5 µM and 5 μM was used, an increase in the effect was observed for both cell types compared to the sum of the individual effects of the substances used (14% and 22% in the case of HeLa cells and 15% and 19% in the case of glioma cells), which clearly indicates the manifestation of a synergism.

Next, experiments were conducted to study the effect of the combined use of such chemotherapeutic agents as doxorubicin (Dx), cyclophosphamide (Cp), or fluorouracil (Fu) with heterocyclic compounds at a final concentration of 200 µM. Data on HeLa and glioma C6 cell viability are presented in Figures S5 and S6, as well as in Tables S8 and S9.

Among these drugs, cyclophosphamide taken at a dose of 25 µM is comparable in strength to carboplatin at the concentrations studied (HeLa cell viability is ~78%, glioma cell viability is ~88%). In turn, fluorouracil and doxorubicin, taken at much lower doses (5 µM and 10 µM, and 25 µM and, 50 µM, respectively), act much more powerfully than carboplatin and cyclophosphamide on both types of cells (HeLa cell viability is ~13–39%, glioma cell viability is ~28–46%).

However, antagonism of the action of fluorouracil/doxorubicin and compounds **5**–**7** was observed on both cell types (see Tables S8 and S9): it was moderate in the case of glioma cells (7–29%) and pronounced in the case of the HeLa cell line (36–67%). The same antagonistic effect (14–20%) was observed when cyclophosphamide at a concentration of 25 µM was used on HeLa cells together with compound **5**–**7**. On the other hand, only weak antagonism was observed for the combination of cyclophosphamide and compound **7** applied to glioma cells (6%), while in the case of derivatives **5** and **6**, a synergistic effect was recorded (8% and 15%).

### 2.4. Quantum Chemical Modeling

Based on the assumption that the effect of the combined action of drugs can be mediated by the formation of non-covalent drug-adjuvant conjugates, we determined structural and electronic changes that occur in the system of two conjugated molecules. The DFT/B3LYP-D3/cc-pvdz/LanL2DZ(Pt,I) level of theory was used for the calculation of the molecules’ optimal geometry, dipole moment, localization, and energy characteristics of frontier molecular orbitals (FMO). Calculations were carried out both for individual compounds and their conjugates, with consideration of the aqueous medium, which simulates the situation in living cells.

**Figure 3:**
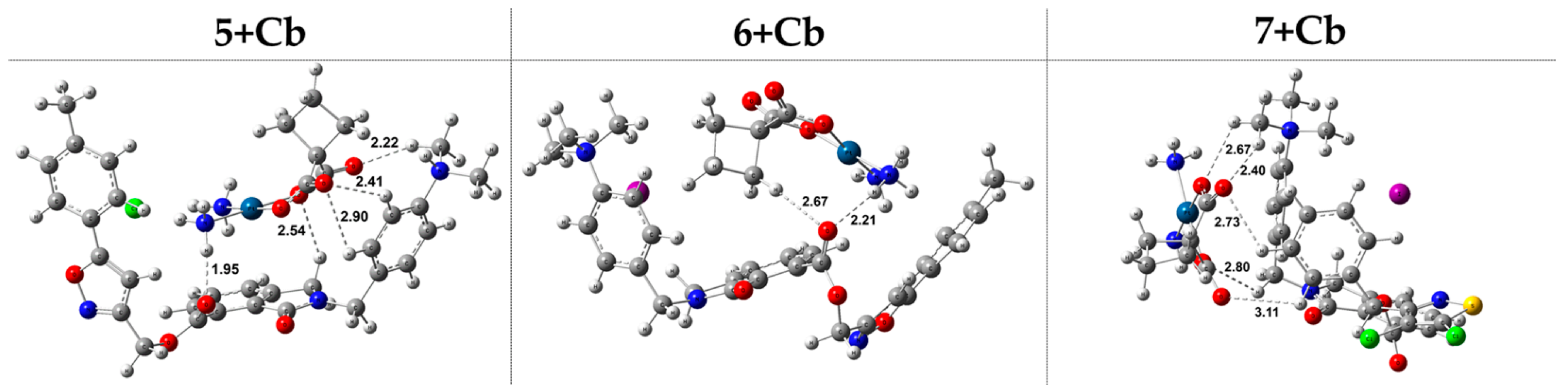
Calculated optimized structures with consideration to aqueous medium for conjugates **5**, **6**, and **7** with carboplatin.

The optimized geometry of the drug+Cb conjugates is presented in Figure 3, and the energy characteristics of the conjugates formation from the initial components are shown in Table 4. The latter (∆E_f_, kcal/mol) were calculated using the following formula: ∆E_f_ = Σ[E_f products_] − Σ[E_f reactants_]. It follows from this calculation data that drug molecules (Cb) and derivatives **5**–**7** form conjugates with shortened interatomic distances due to non-covalent interactions caused by hydrogen and van der Waals bonds (Figure 3).

According to calculation data, despite the similarity in the structure of quaternized derivatives **5**–**7**, the structure of their conjugates with carboplatin differs significantly (Figure 3). The stability of the conjugates is quite close, as indicated by the similar energies of their formation (Table 4), but their existence is realized due to different interactions.

Thus, hydrochloride **5** forms the most stable conjugate with carboplatin due to the short (1.95 Å) and strong N–H···O=C hydrogen bond between the ester group of compound **5** and ammonia ligand of carboplatin, as well as several unconventional C–H···O hydrogen bonds between the aromatic/aliphatic protons of the isoindole moiety and the oxygen atoms of carboplatin (interatomic distances 2.22–2.90 Å). It is noteworthy that one of these unconventional C–H···O hydrogen interactions is comparable in the interatomic distance to the classical N–H···O=C hydrogen bond that occurs during the formation of the carboplatin conjugate with iodomethylate **6** between the ammonia ligand of carboplatin and the ester group of derivative **6**. The latter one is also stabilized by an additional unconventional C–H···O hydrogen interaction (interatomic distance 2.67 Å) between the same ester group and the proton of the C–H bond of the cyclobutane system of carboplatin. This conjugate appeared to be the least stable, since the conjugate of compound **7** with carboplatin, although it did not contain traditional hydrogen bonds, was somewhat more effectively stabilized due to five C–H···O unconventional hydrogen interactions between the oxygen atoms of carboplatin and the protons of the methyl, methylene and aromatic CH groups of compound **7** (interatomic distances 2.40–3.11 Å).

As can be seen, the azole part of the 2-[4-(dimethylamino)benzyl]-3-oxoisoindoline-4-carboxylic acid esters does not participate in the formation of conjugates with carboplatin. However, it, along with such a type of modification as quaternization, dramatically affects the way the conjugate with carboplatin is formed and its stability.

#### 2.4.1. Stability of Conjugates

Unfortunately, the calculated data on the stability of the conjugates do not fully correlate with the biotesting data. Thus, derivative **7** acts in combination with carboplatin more effectively than compound **6**, but this is not reflected in the magnitude of the synergistic effect, since the intrinsic antitumor action of derivative **7** is greater than that of compound **6**. On HeLa cells, the synergistic effect of compound **7** is slightly higher than that of compound **6**, but on glioma C6 cells, the opposite is observed. Moreover, despite the much greater stability of the Cb+**5** conjugate compared to the others, the antitumor effect of derivative **5** in combination with carboplatin is, on average, not significantly different from the effect of the Cb+**6** or Cb+**7** combinations, and the synergistic effect for the Cb+**5** combination is observed mostly on HeLa cells.

The above indicates the absence of causation between the stability of carboplatin conjugates with the studied compounds and their biological action. However, this does not indicate the absence of a relationship with other parameters of these conjugates.

#### 2.4.2. FMO Energies

Frontier molecular orbital (FMO) energies and dipole moments are widely recognized as critical descriptors in assessing a molecule’s biological activity [22,23,24]. Within FMO theory, the key parameter is the energy difference between the highest occupied molecular orbital (HOMO) and the lowest unoccupied molecular orbital (LUMO), commonly referred to as the energy gap (ΔE). The values computed at the DFT/B3LYP-D3/cc-pVDZ/LanL2DZ(Pt,I) level are summarized in Table 5. Across all conjugates, a notable decrease in ΔE is observed (3.87–4.10 eV) compared to carboplatin (5.41 eV). This reduction suggests enhanced chemical reactivity and a potentially greater affinity for biological targets. From this point of view, the conjugate Cb+**6** should be the most bioactive, since its energy gap has the smallest value (3.87 eV). However, the best biological effect is observed for the conjugate Cb+**7**, with the largest energy gap (4.10 eV). Therefore, this characteristic is also not a key factor determining the biological properties of the compounds studied.

**Figure 4:**
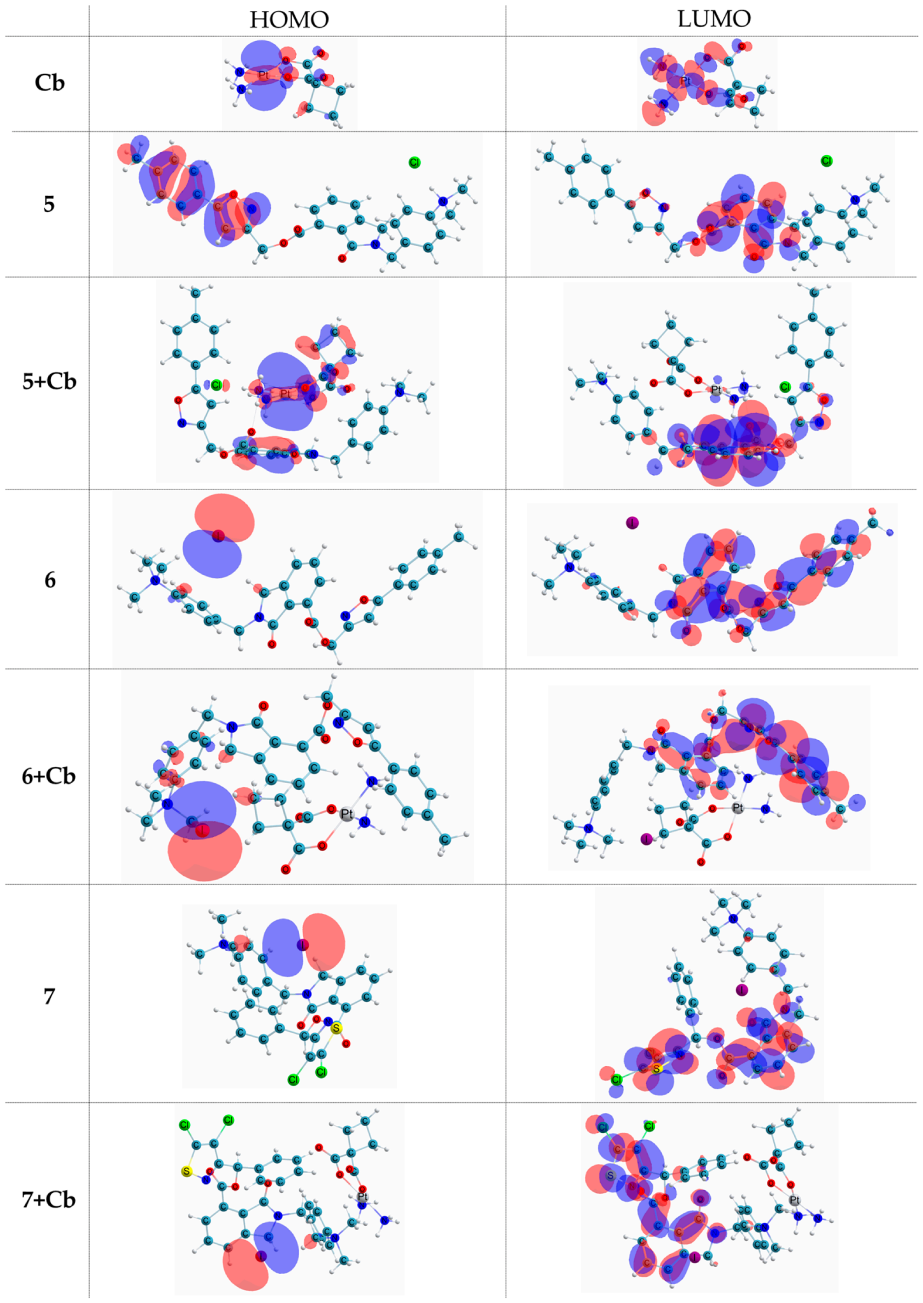
Localization of HOMO and LUMO in the form of 3D isosurfaces for **5**, **6**, **7**, carboplatin, and their conjugates.

Beyond the energies of FMO, another important thing that can influence the bioactivity of compounds is the localization of HOMO and LUMO. Its establishment is important to determining preferred directions and regions of the molecule for attack by nucleophiles and electrophiles. The results of calculating the localization of FMO for derivatives **5**–**7**, carboplatin, and their conjugates are shown in the form of 3D isosurfaces (Figure 4).

The HOMO of carboplatin is distributed on the platinum atom and on the oxygen atoms. The LUMO is also located on the platinum atom and occupies two amino groups and oxygen atoms. For structure **5**, the HOMO is localized on the isoxazoline side, while the LUMO is primarily on the indole, along with the adjacent oxygen atoms. In Cb+**5** conjugate, the HOMO is distributed throughout carboplatin with partial extension to the chlorine atom and the indole. The LUMO is almost entirely on the indole with partial coverage of the isoxazole and Pt atom. The HOMO of **6** is located on the iodine atom. The LUMO fully encompasses the indole and the isoxazole, with the toluene group connected to it via an oxygen atom. The HOMO and LUMO of the carboplatin-containing structure Cb+**6** are mostly the same as those without carboplatin. The HOMO of **6** is totally localized on the iodine atom. The lowest unoccupied molecular orbital is distributed over the indole, the carboxyl group connected to it, and the dichloroisothiazole. The HOMO and LUMO for the structure with carboplatin Cb+**7** are almost the same as without carboplatin: the HOMO is mainly located on the iodine atom, and the LUMO has completely shifted to the indole, the nearby carboxyl group, and the dichloroisothiazole.

So, the conjugation of carboplatin with compounds **5**–**7** results in the displacement of LUMO from the carboplatin to the isoindole derivative. And since it is LUMO that is responsible for the interaction with nucleophilic reagents, which are nitrogenous bases that are part of DNA chains, this interaction will be realized directly through the heterocyclic component of the conjugates, and not through carboplatin. That is, DNA intercalation under the action of carboplatin will be carried out with the direct participation of the added heterocyclic derivative **5**–**7**, and the final result will directly depend on the properties of the added compound.

#### 2.4.3. Global Reactivity Descriptors

Additionally, valuable insights into the reactivity of molecular systems could be obtained from global reactivity descriptors such as electronegativity (χ), chemical hardness and softness (η and S), chemical potential (μ), electrophilicity index (ω), and dipole moment (D). Their theoretical definitions and calculation formulas are well established in the literature [22,23,24]. According to our calculations, the transition from compounds **5**–**7** to their conjugated forms results in notable changes in the above-listed characteristics compared to carboplatin (Table 5). On the one hand, a decrease in chemical potential is observed (from −3.74 eV to up to −4.22 eV in the case of conjugate Cb+**7**), which indicates the stabilization of the system. On the other hand, conjugation leads to an increase in electronegativity (from 3.74 eV to 3.87–4.22 eV), a decrease in chemical hardness (from 2.71 eV to 1.94–2.05 eV), and an increase in chemical softness (from 0.185 eV to 0.244–0.258 eV).

A significant gain in electrophilicity index when moving from carboplatin to its conjugates with compounds **5**–**7** (from 2.586 eV to 3.653–4.430 eV) indicates an increase in the electrophilicity of the system, which may lead to more efficient formation of interstrand cross-links between DNA chains, which underlies the antitumor action of carboplatin [25]. Indeed, biotesting showed an increase in the antitumor activity of the conjugates in comparison with both the original compounds **5**–**7** and carboplatin.

The most conspicuous change was observed for the dipole moment. Thus, if in the case of conjugates Cb+**5** and Cb+**6** it increased by 1.5 times in comparison with carboplatin, then for conjugate Cb+**7**, it reached 1.9 times. As a rule, the dipole moment is associated with the ability of a compound to penetrate cell membranes, and its increase should indicate an increase in the bioavailability of the substance. Here, as can be seen from the results of bioassays, conjugate Cb+**7** showed generally greater antitumor activity than the others, as well as than the original compounds and carboplatin itself.

## 3. Materials and Methods

### 3.1. General Chemistry Section

The reagents and solvents used were of analytical grade, with the content of the main component being more than 99.5%. 5-Phenylisoxazol-3-ylmethanol **2a**, 5-(*p*-tolyl)isoxazol-3-ylmethanol **2b**, 4,5-dichloroisothiazol-3-ylmethanol **2c**, and (4,5-dichloroisothiazol-3-yl)(phenyl)methanol **2d** were synthesized by us according to the procedures developed by us previously [15,16]. Acid **1** is described in the literature as hydrochloride [26]. We synthesized it ourselves in non-quaternized form using a previously described method [27]. The procedure and spectral characteristics are given below.

IR spectra were registered on a Nikolet Protege-460 Fourier transform spectrometer (Nicolet Instrument Corporation, Madison, WI, USA) with samples in KBr pellets.

^1^H and ^13^C NMR spectra were acquired on a Bruker Avance 500 spectrometer (Bruker Corp., MA, Billerica, USA) at500 and 125 MHz, respectively, in CDCl_3_. The residual solvent signals (CDCl_3_, δH 7.26, δC 77.2 ppm; DMSO-*d*_6_, δH 2.50, δC 40.1 ppm) were used as the internal standard. The assignment of signals in the ^13^C NMR spectra was performed using the DEPT technique.

Liquid chromatography–mass spectrometry spectra were recorded on an Agilent 1200 LC-MS system with an Agilent 6410 Triple Quad Mass Selective Detector (Agilent Technologies, Inc., Santa Clara, CA, USA) with electrospray ionization in the positive ion registration mode (MS2 scanning mode). An Agilent ZORBAX Eclipse XDB-C18 (4.6 × 50 mm, 1.8 μm) column was used (Agilent Technologies, Inc., Santa Clara, CA, USA). The mobile phase was MeCN–H_2_O + 0.05% HCO_2_H, with gradient elution from 40 to 90% MeCN in 10 min. A flow rate of 0.5 mL/min was used.

X-ray diffraction experiments were carried out on an automatic four-circle area-detector diffractometer with MoKα radiation Bruker KAPPA APEX II (Bruker Corp., Billerica, MA, USA) [28].

Elemental analysis was performed on a Vario MICRO cube CHNS-analyzer (Elementar Analysensysteme GmbH, Langenselbold, Germany). The halogen content was determined by classical microanalysis according to a modified Pregl method [29].

Melting points were determined on a Kofler bench.

#### 3.1.1. Synthesis of 2-[4-(Dimethylamino)benzyl]-3-oxoisoindoline-4-carboxylic Acid **1**

A mixture of maleic anhydride (20.0 mmol, 1.96 g) and N-[[4-(dimethylamino)phenyl]methyl]-2-furanmethanamine (20.0 mmol, 4.61 g) in toluene (40 mL) was stirred at 25 °C for 1 day. White precipitate was filtered off, washed with toluene (2 × 30 mL), and dried at 80–90 °C for 3 h to constant weight (5.71 g, 17.4 mmol, 87%). This intermediate product was found to be virtually free of impurities, as determined by NMR spectroscopy. Further fine powder of the product (3.28 g, 10.0 mmol) was added to hot 85% phosphoric acid (30 mL) in small portions under constant stirring. The resulting transparent solution was heated at 80 °C for 1 h. The reaction mixture was cooled and poured into crushed ice (~400 mL). The obtained mixture was neutralized with a saturated aqueous ammonia solution until persistent turbidity was observed (pH ~ 7–8), and then left to stand overnight at room temperature. The resulting crystals were collected, washed with cold water (3 × 30 mL) to a neutral reaction in the wash water, and air-dried to constant weight. The product, a pale pink powder, was subsequently recrystallized from an *i*PrOH–DMF mixture with the addition of activated charcoal. The purified crystals were filtered off, washed with water (2 × 15 mL), and air-dried to constant weight to give the target compound.

White powder; yield 1.22 g (39%); mp 206–207 °C; IR (KBr) ν 3436, 2924, 2857, 2806, 1874, 1702, 1610, 1589, 1525, 1506, 1436, 1350, 1285, 1191, 810, 746, 615 cm^−1^; ^1^H NMR (DMSO-*d*_6_, 500 MHz) *δ* 2.87 (6H, s, 2CH_3_), 4.56 (2H, s, CH_2_), 4.72 (2H, s, CH_2_), 6.72 (2H_Ar_, br. d, *J* = 8.8 Hz), 7.21 (2H_Ar_, br. d, *J* = 8.8 Hz), 7.80 (1H_Ar_, t, *J* = 7.6 Hz), 7.87 (1H_Ar_, d, *J* = 7.6 Hz), 8.15 (2H_Ar_, d, *J* = 7.6 Hz) ppm, 16.04 (1H, br. s, CO_2_H); ^13^C NMR (DMSO-*d*_6_, 125 MHz) *δ* 40.1 (2CH_3_), 46.0 (1CH_2_), 50.5 (1CH_2_), 112.4 (2CH_Ar_), 127.8 (1CH_Ar_), 129.3 (2CH_Ar_), 131.7 (1CH_Ar_), 132.2 (1CH_Ar_), 122.9, 128.4, 129.1, 143.1, 150.1, 164.9, 168.5 (7C_quat._) ppm.

#### 3.1.2. General Procedure for the Synthesis of Isoindoline Carboxylic Acid Esters **3**, **4**

N,N′-Dicyclohexylcarbodiimide (DCC) (0.8 mmol, 0.17 g) was added to a pre-cooled (~0 °C) solution of the 2-[4-(dimethylamino)benzyl]-3-oxoisoindoline-4-carboxylic acid **1** (0.8 mmol, 0.25 g), corresponding alcohol (1.6 mmol), and 4-dimethylaminopyridine (DMAP) (0.08 mmol, 0.01 g) in dry dichloromethane (DCM) (5 mL). The reaction mixture was stirred at 0 °C for 5 min and at room temperature until the reaction was complete (18–24 h, TLC control, SiO_2_ plates, eluent—EtOAc). The resulting precipitate was filtered off, and the filtrate was washed with water (3 × 10 mL) and saturated NaCl solution (1 × 10 mL) and dried over Na_2_SO_4_. The drying agent was filtered off, and the solution was evaporated by 2/3 and filtered again through a thin layer of silica gel to remove residual dicyclohexylurea. The filtrate was evaporated to dryness; the resulting precipitate was boiled in methanol (1 mL), filtered off after cooling, washed with cold methanol (2 × 0.5 mL), and dried in air.

(5-Phenylisoxazol-3-yl)methyl 2-(4-(dimethylamino)benzyl)-3-oxoisoindoline-4-carboxylate (**3a**): white solid; yield 63%; mp 163–165 °C; IR (KBr) ν 3452, 3354, 3128, 2935, 2807, 1739, 1686, 1615, 1607, 1526, 1452, 1435, 1280, 1133, 774, 696 cm^−1^; ^1^H NMR (CDCl_3_, 500 MHz) *δ* 2.90 (6H, s, 2CH_3_), 4.24 (2H, s, CH_2_), 4.70 (2H, s, CH_2_), 5.57 (2H, s, CH_2_), 6.65 (2H_Ar_, d, *J* = 7.8 Hz), 6.99 (1H, CH_isox._, s), 7.20 (2H_Ar_, d, *J* = 7.8 Hz), 7.45 (4H_Ar_, m), 7.52 (1H_Ar_, t, *J* = 7.3 Hz), 7.64 (1H_Ar_, d, *J* = 7.1 Hz), 7.81 (2H_Ar_, d, *J* = 6.4 Hz) ppm; ^13^C NMR (CDCl_3_, 125 MHz) *δ* 40.7 (2CH_3_), 46.1 (1CH_2_), 49.0 (1CH_2_), 59.1 (1CH_2_), 100.1 (1CH_isox._), 112.7 (2CH_Ar_), 125.6 (1CH_Ar_), 126.0 (2CH_Ar_), 128.0 (1CH_Ar_), 129.1 (1CH_Ar_), 129.6 (2CH_Ar_), 130.3 (1CH_Ar_), 131.0 (1CH_Ar_), 124.4, 127.5, 129.6, 130.6, 142.4, 150.3, 160.6, 166.0, 167.0, 170.5 (10C_quat._) ppm; MS *m*/*z* (*I*_rel_, %) 468.2 [*M+H*]^+^ (64), 490.2 [*M+Na*]^+^ (22), 957.4 [2*M+Na*]^+^ (100); Anal. calcd. for C_28_H_25_N_3_O_4_ (467.52): C, 71.93; H, 5.39; N, 8.99%. Found: 71.87; H, 5.32; N, 9.11%.

(5-(*p*-tolyl)isoxazol-3-yl)methyl 2-(4-(dimethylamino)benzyl)-3-oxoisoindoline-4-carboxylate (**3b**): white solid; yield 56%; mp 214–216 °C; IR (KBr) ν 3452, 3352, 3119, 2935, 2806, 1740, 1685, 1609, 1526, 1452, 1434, 1281, 1132, 1175, 801 cm^−1^; ^1^H NMR (CDCl_3_, 500 MHz) *δ* 2.39 (3H, s, CH_3_), 2.91 (6H, s, 2CH_3_), 4.25 (2H, s, CH_2_), 4.71 (2H, s, CH_2_), 5.56 (2H, s, CH_2_), 6.65 (2H_Ar_, d, *J* = 8.4 Hz), 6.92 (1H, CH_isox._, s), 7.21 (2H_Ar_, d, *J* = 8.4 Hz), 7.25 (2H_Ar_, d, *J* = 8.4 Hz), 7.46 (1H_Ar_, d, *J* = 7.6 Hz), 7.52 (1H_Ar_, t, *J* = 7.5 Hz), 7.64 (1H_Ar_, t, *J* = 7.4 Hz), 7.70 (2H_Ar_, d, *J* = 8.0 Hz) ppm; ^13^C NMR (CDCl_3_, 125 MHz) *δ* 21.6 (1CH_3_), 40.7 (2CH_3_), 46.1 (1CH_2_), 49.1 (1CH_2_), 59.1 (1CH_2_), 99.5 (1CH_isox._), 112.7 (2CH_Ar_), 125.5 (1CH_Ar_), 126.0 (2CH_Ar_), 128.0 (1CH_Ar_), 129.6 (2CH_Ar_), 129.8 (2CH_Ar_), 131.0 (1CH_Ar_), 124.4, 124.8, 129.7, 130.6, 140.6, 142.4, 150.3, 160.6, 166.0, 167.0, 170.8 (11C_quat._) ppm; MS *m*/*z* (*I*_rel_, %) 482.2 [*M+H*]^+^ (50), 504.2 [*M+Na*]^+^ (27), 985.4 [2*M+Na*]^+^ (100); Anal. calcd. for C_29_H_27_N_3_O_4_ (481.55): C, 72.33; H, 5.65; N, 8.73%. Found: C, 72.27; H, 5.50; N, 8.88%.

4,5-Dichloroisothiazol-3-ylmethyl 2-(4-(dimethylamino)benzyl)-3-oxoisoindoline-4-carboxylate (**4a**): white solid; yield 57%; mp 167–169 °C; IR (KBr) ν 3429, 3342, 2927, 1752, 1725, 1687, 1610, 1522, 1291, 1279, 1136, 971, 796, 743, 607 cm^−1^; ^1^H NMR (CDCl_3_, 500 MHz) *δ* 2.92 (6H, s, 2CH_3_), 4.21 (2H, s, CH_2_), 4.67 (2H, s, CH_2_), 5.56 (2H, s, CH_2_), 6.67 (2H_Ar_, d, *J* = 8.7 Hz), 7.18 (2H_Ar_, d, *J* = 8.6 Hz), 7.45 (1H_Ar_, d, *J* = 7.4 Hz), 7.51 (1H_Ar_, t, *J* = 7.6 Hz), 7.66 (2H_Ar_, d, *J* = 7.5 Hz) ppm; ^13^C NMR (CDCl_3_, 125 MHz) *δ* 40.7 (2CH_3_), 46.1 (1CH_2_), 49.0 (1CH_2_), 62.3 (1CH_2_), 112.7 (2CH_Ar_), 125.5 (1CH_Ar_), 128.1 (1CH_Ar_), 129.7 (2CH_Ar_), 130.9 (1CH_Ar_), 124.5, 129.6, 129.8, 130.7, 142.4, 148.5, 150.3, 161.2, 165.9, 166.3 (10C_quat._) ppm; MS (for ^35^Cl) *m*/*z* (*I*_rel_, %) 476.0 [*M+H*]^+^ (49), 973.1 [2*M+Na*]^+^ (63); Anal. calcd. for C_22_H_19_Cl_2_N_3_O_3_S (476.37): C, 55.47; H, 4.02; Cl, 14.88; N, 8.82; S, 6.73%; Found: C, 55.40; H, 3.89; Cl, 14.90; N, 8.94; S, 6.59%.

(4,5-Dichloroisothiazol-3-yl)(phenyl)methyl 2-(4-(dimethylamino)benzyl)-3-oxoisoindoline-4-carboxylate (**4b**): white solid; yield 62%; mp 216–218 °C; IR (KBr) ν 3450, 3358, 2918, 1746, 1688, 1613, 1527, 1279, 1134, 982, 750, 701, 610 cm^−1^; ^1^H NMR (CDCl_3_, 500 MHz) *δ* 2.92 (6H, s, 2CH_3_), 4.19 (2H, s, CH_2_), 4.65 (2H, m, CH_2_), 6.66 (2H_Ar_, d, *J* = 8.5 Hz), 7.18 (2H_Ar_, d, *J* = 8.5 Hz), 7.23 (1H, s, CH), 7.39 (3H_Ar_, m), 7.43 (1H_Ar_, d, *J* = 7.6 Hz), 7.51 (1H_Ar_, t, *J* = 7.6 Hz), 7.60 (2H_Ar_, d, *J* = 7.2 Hz), 7.76 (1H_Ar_, d, *J* = 7.5 Hz) ppm; ^13^C NMR (CDCl_3_, 125 MHz) *δ* 40.7 (2CH_3_), 46.1 (1CH_2_), 48.9 (1CH_2_), 74.4 (1CH), 112.7 (2CH_Ar_), 125.5 (1CH_Ar_), 128.4 (2CH_Ar_), 128.4 (1CH_Ar_), 128.8 (2CH_Ar_), 129.1 (1CH_Ar_), 129.7 (2CH_Ar_), 130.8 (1CH_Ar_), 122.4, 124.6, 129.6, 130.9, 135.6, 142.5, 148.6, 150.3, 163.9, 165.2, 165.7 (11C_quat._) ppm; MS (for ^35^Cl) *m*/*z* (*I*_rel_, %) 552.1 for [*M+H*]^+^ (72), 574.1 [*M+Na*]^+^ (25), 1125.2 [2*M+Na*]^+^ (60); Anal. calcd. for C_28_H_23_Cl_2_N_3_O_3_S (552.47): C, 60.87; H, 4.20; Cl, 12.83; N, 7.61; S, 5.80%; Found: C, 60.82; H, 4.13; Cl, 12.89; N, 7.74; S, 5.75%.

#### 3.1.3. Procedure for the Synthesis of Hydrochloride **5**

The initial ester **1** (0.77 mmol, 0.3 g) was dissolved in dry dichloromethane (30 mL); the resulting solution was diluted with dry ether (30 mL), and dry hydrogen chloride was bubbled through the reaction mixture. The resulting precipitate was filtered on a fine-pored Schott filter, washed with diethyl ether (1 × 5 mL), and dried in vacuo.

N,N-Dimethyl-4-((1-oxo-7-(((5-(*p*-tolyl)isoxazol-3-yl)methyl)carbamoyl)isoindolin-2-yl)methyl)benzeneammonium chloride (**5**): white solid, yield 76%; mp 185–187 °C. IR (KBr) ν 3424, 2921, 2854, 2499, 2429, 1738, 1727, 1692, 1612, 1590, 1574, 1514, 1503, 1481, 1467, 1451, 1434, 1408, 1374, 1324, 1287, 1206, 1169, 1132, 1053, 1022, 994, 948, 771, 750, 731, 696 cm^−1^; ^1^H NMR (CDCl_3_, 500 MHz) *δ* 2.36 (3H, s, CH_3_), 3.13 (6H, s, 2CH_3_), 4.33 (2H, s, CH_2_), 4.80 (2H, s, CH_2_), 5.52 (2H, s, CH_2_), 6.78 c (1H, s, CH_isox._), 7.22 (2H_Ar_, d, *J* = 8.2 Hz), 7.43 (2H_Ar_, br. s), 7.56 (1H_Ar_, br. s), 7.62 (2H_Ar_, d, *J* = 7.6 Hz), 7.67 (1H_Ar_, m), 7.72 (1H_Ar_, d, *J* = 4.3 Hz), 7.75 (2H_Ar_, br. s), 13.82 (1H, br. s, NH) ppm; ^13^C NMR (CDCl_3_, 126 MHz) *δ* 21.6 (1CH_3_), 45.9 (1CH_2_), 46.8 (2CH_3_), 49.5 (1CH_2_), 59.1 (1CH_2_), 99.1 (1CH_isox._), 121.5 (2CH_Ar_), 125.7 (1CH_Ar_), 125.8 (2CH_Ar_), 128.8 (1CH_Ar_), 129.8 (2CH_Ar_), 130.3 (2CH_Ar_), 131.6 (1CH_Ar_), 124.4, 129.3, 129.8, 139.4, 140.8, 142.2, 142.5, 160.1, 166.4, 166.5, 170.8 (11C_quat._) ppm; MS *m*/*z* (*I*_rel_, %) 482.3 [*M–Cl^−^*]^+^ (100); Anal. calcd. for C_29_H_28_ClN_3_O_4_ (518.01): C 67.24; H 5.45; Cl 6.84; N 8.11%; Found: C 67.19; H 5.40; Cl 6.81; N 8.35%.

#### 3.1.4. General Procedure for the Synthesis of Iodomethylates **6**, **7**

The initial ester **3b** or **4b** (0.77 mmol) was dissolved in dry dichloromethane (30 mL); iodomethane (1 mL, 16.06 mmol) was added, and the reaction mixture was stirred in the dark for 48 h. The solvent was removed, and the crude product was washed with a mixture of chloroform and diethyl ether (3:1; 2 × 4 mL), then diethyl ether (5 mL), and then dried in vacuo.

4-((1-Oxo-7-(((5-(*p*-tolyl)isoxazol-3-yl)methyl)carbamoyl)isoindolin-2-yl)methyl)-N,N,N-threemethylbenzeneammonium iodide (**6**): pale yellow solid, yield 89%; mp 169–170 °C. IR (KBr) ν 3460, 3308, 3073, 1739, 1687, 1618, 1459, 1434, 1285, 1261, 1201, 1138, 820, 751, 614 cm^−1^; ^1^H NMR (DMSO-*d*_6_, 500 MHz) *δ* 2.36 (3H, s, CH_3_), 3.59 (9H, s, 3CH_3_), 4.50 (2H, s, CH_2_), 4.81 (2H, s, CH_2_), 5.47 (2H, s, CH_2_), 7.16 (1H, s, CH_isox._), 7.35 (2H_Ar_, d, *J* = 8.0 Hz), 7.55 (2H_Ar_, d, *J* = 9.0 Hz), 7.70 (2H_Ar_, m), 7.76 (3H_Ar_, m), 7.94 (2H_Ar_, d, *J* = 9.0 Hz) ppm; ^13^C NMR (DMSO-*d*_6_, 126 MHz) *δ* 21.6 (1CH_3_), 45.3 (1CH_2_), 50.1 (1CH_2_), 57.0 (3CH_3_), 59.0 (1CH_2_), 100.1 (1CH_isox._), 121.4 (2CH_Ar_), 126.2 (2CH_Ar_), 126.7 (1CH_Ar_), 127.6 (1CH_Ar_), 129.8 (2CH_Ar_), 130.4 (2CH_Ar_), 132.1 (1CH_Ar_), 124.5, 129.3, 129.5, 140.1, 141.1, 143.4, 146.9, 161.0, 166.2, 167.0, 170.3 (11C_quat._) ppm; MS *m*/*z* (*I*_rel_, %) 496.4 [*M–*I]^+^ (100); Anal. calcd. for C_30_H_30_IN_3_O_4_ (623.49): C 57.79; H 4.85; I 20.35; N 6.74%; Found: C 57.61; H 4.74; I 20.47; N 6.82%.

4-((1-Oxo-7-(((4,5-dichloroisothiazol-3-yl)(phenyl)methyl)carbamoyl)isoindolin-2-yl)methyl)-N,N,N-threemethylbenzeneammonium iodide (**7**): pale yellow solid, yield 84%; mp 174–176 °C. IR (KBr) ν 3439, 3035, 2934, 1735, 1717, 1687, 1606, 1506, 1492, 1456, 1434, 1408, 1385, 1317, 1286, 1211, 1192, 1171, 1145, 1092, 1011, 979, 957, 939, 860, 848, 794, 749, 716, 698, 611 cm^−1^; ^1^H NMR (DMSO-*d*_6_, 500 MHz) *δ* 3.58 s (9H, 3CH_3_), 4.47 s (2H, CH_2_), 4.76 q (2H, CH_2_, *J* = 15.4 Hz), 7.07 s (1H, CH), 7.42 m (2H_Ar_), 7.53 dd (4H_Ar_, *J* = 7.3, 4.0 Hz), 7.72 m (3H_Ar_), 7.93 d (2H_Ar_, *J* = 8.8 Hz) ppm; ^13^C NMR (DMSO-*d*_6_, 126 MHz) *δ* 45.3 (1CH_2_), 50.1 (1CH_2_), 57.0 (3CH_3_), 74.4 (1CH), 121.4 (2CH_Ar_), 126.9 (1CH_Ar_), 127.8 (1CH_Ar_), 128.3 (2CH_Ar_), 129.3 (2CH_Ar_), 129.6 (1CH_Ar_), 129.8 (2CH_Ar_), 132.2 (2CH_Ar_), 79.8, 129.2, 129.4, 135.7, 140.1, 143.5, 146.9, 149.1, 163.7, 165.9, 166.0 (11C_quat._) ppm; MS (for ^35^Cl) *m*/*z* (*I*_rel_, %) 566.1 [*M−*I]^+^ (100); Anal. calcd. for C_29_H_26_Cl_2_IN_3_O_3_S (694.41): C, 50.16; H, 3.77; Cl, 10.21; I, 18.28; N, 6.05; S, 4.62%; Found: C, 50.11; H, 3.64; Hal, 28.65; N, 6.25; S, 4.54%.

### 3.2. In Vitro Biological Assays

#### 3.2.1. Testing of Anticancer Activity and Synergistic Effect in Compositions with First-Line Antitumor Drugs

Cell lines. This research was carried out on HeLa (cervical cancer, human) and C6 glioma (rat) cell lines from the Laboratory of Immunology and Cell Biotechnology of the Research Institute of Hygiene, Toxicology, Epidemiology, Virology and Microbiology (description of the collection: https://belriem.by/o-tsentre/otdely-i-laboratorii/laboratoriya-immunologii-i-kletochnoy-biotekhnologii/ (accessed on 23 April 2026)). Information about the Collection is included in the union catalog of the Russian Collection of Cell Cultures of the Institute of Cytology of the Russian Academy of Sciences https://cellcollection.ru/catalog/ (accessed on 23 April 2026).

Sample preparation. The solutions (0.5 mM stock) of the test samples were prepared in 0.9% isotonic sodium chloride solution. Further dilutions were made in isotonic sodium chloride solution. The solutions were added to the wells of the plate with growing cells in a volume ratio of 1:9 (test compounds/medium with cells).

Antitumor drugs: carboplatin (*Fresenius Kabi*, Friedberg, Germany), doxorubicin (*RUPE* “*Belmedpreparaty*”, Minsk, Belarus), cyclophosphamide (*RUPE* “*Belmedpreparaty*”, Minsk, Belarus), and fluorouracil (*RUPE* “*Belmedpreparaty*”, Minsk, Belarus).

Conducting experiments. The cells were seeded into wells of 96-well plates (Corning, Kennebunk, ME, USA) in Dulbecco’s modified Eagle medium 5648 (“*Sigma-Aldrich*”, Saint Louis, MO, USA) supplemented with 10% fetal bovine serum (“*Sigma-Aldrich*”, Burlington, MA, USA) and antibiotics (penicillin, streptomycin, amphotericin B, “*Biological Industries*”, Cromwell, CT, USA). After 24 h, the test compounds at a final concentration of 100 or 200 μM and/or antitumor drugs at a final concentration of 5–50 μM were added to the wells. Isotonic sodium chloride solution was added as a control. The cells were cultured for 48 h at 37 °C and 5% CO_2_. Then, the cell samples were analyzed using the standard MTT tests with 3-(4,5-dimethylthiazol-2-yl)-2,5-diphenyl tetrazolium bromide (“Glentham Life Sciences”, Corsham, UK).

Statistical processing. The resulting digital material was processed by methods of variation statistics using the Excel and Statistica 7 software packages. The data are presented as the mean and its standard error. Differences between series were considered significant at a significance level of *p* < 0.05 according to Student’s *t*-test.

#### 3.2.2. Cytotoxicity Testing

The experiments were conducted on 2-day-old larvae of the marine crustacean *Artemia salina* (Leach) under in vitro cultivation conditions. The larvae were grown by immersing the eggs of the marine crustacean in artificial seawater and incubating them for 48 h at a temperature of 37 °C. The concentrations of the tested substances were 100 μg/mL, 10 μg/mL, and 1 μg/mL; the experiments were performed in 3 repetitions. Ten 2-day-old larvae of the marine crustacean *Artemia salina* were placed in each vial with a sample. Then, all the vials were left at room temperature in the light for 24 h. After 24 h, the surviving and dead larvae were counted. Then, using the obtained data on the upper and lower toxic limits, the half-toxic dose of the sample was calculated. Control—DMSO in equivolume amounts. The test was conducted using the comparison drug—dactinomycin (actinomycin D)—which has antitumor (cytotoxic) activity. Statistical processing of the results was performed using the FIN computer program.

### 3.3. Quantum Chemical Methods

The Orca 5.0.3 package [30] was employed to perform the DFT calculations, while the visualization of the obtained results was carried out using ChemCraft (version 1.8, build 809bt) and Avogadro (version 1.2.0) software [31,32]. The computations were conducted within the framework of the DFT-D3 dispersion correction method [33], which, as demonstrated in numerous studies [34,35,36], is well suited to describing both inter- and intramolecular long-range dispersion interactions. The B3LYP hybrid functional, combining Becke’s three-parameter exchange functional [37] with the correlation functional of Lee et al. [38], was applied, since this functional is known to provide high accuracy when compared with experimental data for systems involving intermolecular interactions [39,40].

For the basis sets, the aug-cc-pVDZ correlation-consistent polarized valence double-zeta set developed by Dunning, augmented with diffuse functions, was employed. The inclusion of diffuse functions allows for an accurate description of long-range interactions such as van der Waals forces and noncovalent interactions, including hydrogen bonding [41]. For platinum and iodine atoms, the LANL2DZ basis set with an effective core potential (ECP) was used [42,43,44].

To model the solvent environment, the polarizable continuum model (PCM) was adopted, where the solvent is treated as a continuous dielectric medium [45]. This approach is based on the self-consistent reaction field (SCRF) method, which accounts for solvent polarization in terms of electrostatic potential. Unless otherwise specified, all subsequent discussions are based on this solvation model. No symmetry constraints were applied during the geometry optimizations, and the identification of energy minima was confirmed by subsequent frequency calculations.

## 4. Conclusions

Guided by the data on the bioactivity and ability of some azole derivatives of alkaloids to enhance the effect of antitumor drugs, we obtained a number of new esters of the isoindole series. The target substances—isoxazole and isothiazole 2-[4-(dimethylamino)benzyl]-3-oxoisoindoline-4-carboxylates—were synthesized by the carbodiimide method and quaternized for better solubility.

Biotesting showed that the obtained compounds are moderately cytotoxic and, when used alone, have a relatively weak ability to suppress the viability of tumor cells. However, in combination with carboplatin, they demonstrated a synergistic effect when the drug was taken in low doses (0.5–5 μM); interestingly, at higher concentrations, any effect was generally absent. In combination with doxorubicin, cyclophosphamide, or fluorouracil, antagonism was mainly observed, more pronounced on HeLa cells and less so on glioma C6 cells.

The difference in the observed effects, as well as their dose dependence, in all likelihood is directly related to the mechanism of action of specific chemotherapy drugs. It was hypothesized that this mechanism is influenced by the specific interaction between the chemotherapeutics and azole derivatives. Using quantum chemical calculations, it was evaluated how the presence of a heterocyclic additive affects the state of the chemotherapeutic drug molecule. This approach was justified, since a significant redistribution of the electron density was discovered, as well as an increase in electrophilicity and dipole moment, which ultimately indicates an increase in reactivity. Unfortunately, it does not explain the dependence of the observed effect on the dose of the chemotherapy drug, so this is a subject for further study.

## Figures and Tables

**Table 1:** Cytotoxicity assay results for the derivatives obtained.

Substance	(LD_50_, μM)
**3a**	204.9
**3b**	192.3
**4a**	215.0
**4b**	200.0
**5**	133.6
**6**	119.0
**7**	111.3
Dactinomycin (actinomycin D)	35.6

**Table 2:** Decrease in HeLa cell viability due to the application of carboplatin (0–40 μM), compounds **5**–**7**, and their compositions.

Cb Dose	Cb Action, %	Compound Tested (200 μM)	Joint Action, %	Σ	Observed Effect
0 µM	–	**5**	20.2	–	–
		**5** (100 μM)	16.4		
		**6**	34.8		
		**6** (100 μM)	29.9		
		**7**	58.1		
		**7** (100 μM)	56.1		
0.5 µM	–	**5**	22.4	20.2	Syn (~2%)
		**6**	38.7	34.8	Syn (~4%)
		**7**	72.4	58.1	Syn (~14%)
5 µM	3.3	**5**	46.5	23.5	Syn (~23%)
		**6**	54.6	38.1	Syn (~17%)
		**7**	83.6	61.4	Syn (~22%)
10 µM	10.2	**5**	34.6	30.4	Syn (~4%)
		**6**	31.8	45.0	Ant (~13%)
		**7**	62.9	68.3	Ant (~5%)
20 µM	15.3	**5**	50.1	35.5	Syn (~15%)
		**6**	33.6	50.1	Ant (~17%)
		**7**	54.5	73.4	Ant (~20%)
40 µM	15.6	**5**	49.1	35.8	Syn (~13%)
		**5** (100 μM)	30.7	32.0	Ant (~20%)
		**6**	42.7	50.4	Ant (~1%)
		**6** (100 μM)	43.1	45.5	Ant (~2%)
		**7**	60.6	73.7	Ant (~13%)
		**7** (100 μM)	57.3	71.7	Ant (~14%)

Σ—summarized effect of the drug and compound itself; Ant—antagonism; Syn—synergism.

**Table 3:** Decrease in glioma C6 cell viability due to the application of carboplatin (0–40 μM), compounds **5**–**7,** and their compositions.

Cb Dose	Cb Action, %	Compound Tested (200 μM)	Joint Action, %	Σ	Observed Effect
0 µM	–	**5**	38.2	–	–
		**5** (100 μM)	26.1		
		**6**	22.1		
		**6** (100 μM)	26.9		
		**7**	34.9		
		**7** (100 μM)	41.8		
0.5 µM	–	**5**	41.5	38.2	Syn (~3%)
		**6**	43.6	22.1	Syn (~22%)
		**7**	49.6	34.9	Syn (~15%)
5 µM	0.7	**5**	48.6	38.9	Syn (~10%)
		**6**	52.7	22.8	Syn (~30%)
		**7**	54.5	35.6	Syn (~19%)
10 µM	7.1	**5**	42.4	45.3	Ant (~3%)
		**6**	20.5	29.2	Ant (~9%)
		**7**	44.9	42.0	Syn (~3%)
20 µM	12.2	**5**	54.7	50.4	Syn (~4%)
		**6**	33.3	34.3	Ant (~1%)
		**7**	48.1	47.1	Syn (~1%)
40 µM	15.2	**5**	54.9	53.4	Syn (~2%)
		**5** (100 μM)	38.8	41.3	Ant (~3%)
		**6**	34.0	37.3	Ant (~3%)
		**6** (100 μM)	38.8	42.1	Ant (~3%)
		**7**	51.7	50.1	Syn (~2%)
		**7** (100 μM)	47.9	57.0	Ant (~9%)

Σ—summarized effect of the drug and compound itself; Ant—antagonism; Syn—synergism.

**Table 4:** Energy characteristics of the conjugate formation process from the initial components **5**, **6**, **7,** and **Cb**.

Conjugate	∆E_f_, kJ/mol	∆E_f_, kcal/mol
**5** **+** **Cb**	−141.9520	−33.9046
**6** **+** **Cb**	−120.5770	−28.7527
**7** **+** **Cb**	−122.8130	−29.2859

**Table 5:** DFT calculated molecular orbital energies and global reactivity descriptor values for **5**, **6**,**7,** **Cb** and their conjugates.

	E_HOMO_, eV	E_LUMO_, eV	∆E, eV	μ, eV	χ, eV	η, eV	S, eV^−1^	ω, eV	D, Db
**5**	−6.50	−1.87	4.63	−4.19	4.19	2.32	0.216	3.792	13.85
**6**	−5.91	−1.90	4.01	−3.91	3.91	2.01	0.249	3.812	13.82
**7**	−5.92	−1.85	4.07	−3.89	3.89	2.04	0.246	3.804	23.78
**Cb**	−6.44	−1.03	5.41	−3.74	3.74	2.71	0.185	2.586	21.34
**5** **+Cb**	−6.23	−2.21	4.02	−4.22	4.22	2.01	0.249	4.430	32.58
**6** **+Cb**	−5.89	−2.02	3.87	−3.96	3.96	1.94	0.258	4.052	32.93
**7** **+Cb**	−5.92	−1.82	4.10	−3.87	3.87	2.05	0.244	3.653	39.50

## Data Availability

The data presented in this study are available in the article or Supplementary Material.

## Supplementary Materials

The following supporting information can be downloaded at https://www.mdpi.com/article/10.3390/molecules31091528/s1: The results of XRD study and raw data of biotesting [46,47,48]. Table S1. Crystal data and structure refinement for compounds **3a** and **3b**; Table S2. Fractional Atomic Coordinates (×10^4^) and Equivalent Isotropic Displacement Parameters (Å^2^ × 10^3^) for 2a and 2b. U_eq_ is defined as 1/3 of the trace of the orthogonalised U_IJ_ tensor; Table S3. Anisotropic Displacement Parameters (Å^2^ × 10^3^) for 2a and 2b. The Anisotropic displacement factor exponent takes the form: −2π^2^[h^2^a*^2^U_11_ + 2hka*b*U_12_ + …]; Table S4. Bond Lengths for **3a** and **3b**; Table S5. Bond Angles for **3a** and **3b**; Table S6. Torsion Angles for **3a** and **3b**; Table S7. Hydrogen Atom Coordinates (Å × 10^4^) and Isotropic Displacement Parameters (Å^2^ × 10^3^) for **3a** and **3b**; Figure S1. Packing diagram of **3a** and **3b**. Dotted lines show π-π interactions; Figure S2. Effect of **5**–**7** (200 µM), carboplatin (0.5–40 µM) and their mixtures on the viability of HeLa cells, * *p* < 0.05 when compared with the control; ** *p* < 0.05 when compared with the effect of carboplatin at the corresponding dose (for series where the combined action of drugs was used); *** *p* < 0.05 when compared with the effect of the corresponding heterocyclic drug (for series where the combined action of drugs was used); Figure S3. Effect of **5**–**7** (200 µM), carboplatin (0.5–40 µM) and their mixtures on the viability of glioma C6 cells, * *p* < 0.05 when compared with the control; ** *p* < 0.05 when compared with the effect of carboplatin at the corresponding dose (for series where the combined action of drugs was used); *** *p* < 0.05 when compared with the effect of the corresponding heterocyclic drug (for series where the combined action of drugs was used); Figure S4. Effect of **5**–**7** (100 µM), carboplatin (40 µM) and their mixtures on the viability of Hela (a) and glioma C6 (b) cells, * *p* < 0.05 when compared with the control; ** *p* < 0.05 when compared with the effect of the corresponding heterocyclic drug (for series where the combined action of drugs was used); *** *p* < 0.05 when compared with the effect of carboplatin at the corresponding dose (for series where the combined action of drugs was used); Figure S5. Effect of **5**–**7** (200 µM) in compositions with doxorubicin (5 or 10 µM), cyclophosphamide (25 µM) or fluorouracil (25 or 50 µM) on the viability of Hela cells, * *p* < 0.05 when compared with the control; ** *p* < 0.05 when compared with the effect of the corresponding heterocyclic drug (for series where the combined action of drugs was used); *** *p* < 0.05 when compared with the effect of carboplatin at the corresponding dose (for series where the combined action of drugs was used); Table S8. Decrease in Hela cell viability due to the application of doxorubicin (5 or 10 µM), cyclophosphamide (25 µM) or fluorouracil (25 or 50 µM), compounds **5**–**7** and their compositions; Figure S6. Effect of **5**–**7** (200 µM) in compositions with doxorubicin (5 or 10 µM), cyclophosphamide (25 µM) or fluorouracil (25 or 50 µM) on the viability of glioma C6 cells, * *p* < 0.05 when compared with the control; ** *p* < 0.05 when compared with the effect of the corresponding heterocyclic drug (for series where the combined action of drugs was used); *** *p* < 0.05 when compared with the effect of carboplatin at the corresponding dose (for series where the combined action of drugs was used); Table S9. Decrease in glioma C6 cell viability due to the application of doxorubicin (5 or 10 µM), cyclophosphamide (25 µM) or fluorouracil (25 or 50 µM), compounds **5**–**7** and their compositions.

## References

[1] Chikkula K.V., Raja S. (2017). Isoxazole—A potent pharmacophore. Int. J. Pharm. Pharm. Sci..

[2] Kletskov A.V., Bumagin N.A., Zubkov F.I., Grudinin D.G., Potkin V.I. (2020). Isothiazoles in the design and synthesis of biologically active substances and ligands for metal complexes. Synthesis.

[3] Vashisht K., Sethi P., Bansal A., Bansal P. (2024). Antimicrobial activity of isoxazole derivatives: A brief overview. Vietnam J. Chem..

[4] Li E.K., Tam L.-S., Tomlinson B. (2004). Leflunomide in the treatment of rheumatoid arthritis. Clin. Ther..

[5] Kothare S.V., Kaleyias J. (2008). Zonisamide: Review of pharmacology, clinical efficacy, tolerability, and safety. Expert Opin. Drug Metab. Toxicol..

[6] Minwalla H.D., Wrzesinski P., Desforges A., Caskey J., Wagner B., Ingraffia P., Patterson J.C., Edinoff A.N., Kaye A.M., Kaye A.D. (2021). Paliperidone to Treat Psychotic Disorders. Neurol. Int..

[7] Stahl S.M., Shayegan D.K. (2003). The psychopharmacology of Ziprasidone: Receptor-binding properties and real-world psychiatric practice. J. Clin. Psychiatry.

[8] Yasui-Furukori N., Furukori H., Nakagami T., Saito M., Inoue Y., Kaneko S., Tateishi T. (2004). Steady-state pharmacokinetics of a new antipsychotic agent perospirone and its active metabolite, and its relationship with prolactin response. Ther. Drug Monit..

[9] Potkin V., Pushkarchuk A., Zamaro A., Zhou H., Kilin S., Petkevich S., Kolesnik I., Michels D.L., Lyakhov D.A., Kulchitsky V.A. (2023). Effect of the isotiazole adjuvants in combination with cisplatin in chemotherapy of neuroepithelial tumors: Experimental results and modeling. Sci. Rep..

[10] Kulchitsky V.A., Potkin V.I., Zubenko Y.S., Chernov A.N., Talabaev M.V., Demidchik Y.E., Petkevich S.K., Kazbanov V.V., Gurinovich T.A., Roeva M.O. (2012). Cytotoxic effects of chemotherapeutic drugs and heterocyclic compounds at application on the cells of primary culture of neuroepithelium tumors. Med. Chem..

[11] Kletskov A.V., Potkin V.I., Kolesnik I.A., Petkevich S.K., Kvachonak A.V., Dosina M.O., Loiko D.O., Larchenko M.V., Pashkevich S.G., Kulchitsky V.A. (2018). Synthesis and biological activity of novel comenic acid derivatives containing isoxazole and isothiazole moieties. Nat. Prod. Commun..

[12] Kolesnik I.A., Kletskov A.V., Potkin V.I., Knizhnikov V.A., Zvereva T.D., Kurman P.V., Tokalchik Y.P., Kulchitsky V.A. (2021). Glycylglycine and its morpholide derivatives containing 5-(*p*-tolyl)isoxazole and 4,5-dichloroisothiazole moieties. Russ. J. Org. Chem..

[13] Mukusheva G.K., Jalmakhanbetova R.I., Shaibek A.Z., Nurmaganbetova M.S., Zhasymbekova A.R., Nurkenov O.A., Akishina E.A., Kolesnik I.A., Dikusar E.A., Terpinskaya T.I. (2024). Alkaloid-based isoxazolylureas: Synthesis and effect in combination with anticancer drugs on c6 rat glioma model cells. Molecules.

[14] Speck K., Magauer T. (2013). The chemistry of isoindole natural products. Beilstein J. Org. Chem..

[15] Potkin V.I., Petkevich S.K., Kletskov A.V., Dikusar E.A., Zubenko Y.S., Zhukovskaya N.A., Kazbanov V.V., Pashkevich S.G. (2013). Synthesis of functionally substituted isoxazole and isothiazole derivatives. Russ. J. Org. Chem..

[16] Potkin V.I., Bumagin N.A., Kletskov A.V., Petkevich S.K., Kurman P.V. (2016). Synthesis of functional derivatives of isothiazole and isoxazole basing on (5-arylizoxazol-3-yl)- and (4,5-dichloroisothiazol-3-yl)arylmethanol. Russ. J. Org. Chem..

[17] Kolesnik I.A., Potkin V.I., Grigoriev M.S., Gomila R.M., Nikitina E.V., Zaytsev V.P., Zubkov F.I., Frontera A. (2025). n→π* and Chalcogen bonds in azole-substituted isoindole derivatives: A combined crystallographic and computational study. CrystEngComm.

[18] Hermanson G.T. (2013). Bioconjugate Techniques.

[19] Sarah Q.S., Anny F.C., Misbahuddin M. (2017). Brine shrimp lethality assay. Bangladesh J. Pharmacol..

[20] Jaewon Y., Amorim H., Andrade L., Bueno M.G.S., Crnkovic C.M. (2025). Screening Natural Products Against Artemia salina.

[21] Kumar P., Nagarajan A., Uchil P.D. (2018). Analysis of cell viability by the MTT assay. Cold Spring Harb. Protoc..

[22] Geerlings P. (2022). From density functional theory to conceptual density functional theory and biosystems. Pharmaceuticals.

[23] Flores-Holguín N., Frau J., Glossman-Mitnik D. (2025). Exploring the reactivity landscape of bioactive peptides using conceptual DFT and molecular modeling tools. Comput. Biol. Chem..

[24] Poddar A., Pal R., Patra S.G., Chattaraj P.K., Basak S.C. (2025). Quantitative structure-activity analysis using conceptual DFT and information theory-based descriptors. Mathematical Descriptors of Molecules and Biomolecules.

[25] Noll D.M., Mason T.M., Miller P.S. (2006). Formation and repair of interstrand cross-links in DNA. Chem. Rev..

[26] Li G.B., Abboud M.I., Brem J., Someya H., Lohans C.T., Yang S.Y., Spencer J., Wareham D.W., McDonough M.A., Schofield C.J. (2017). NMR-filtered virtual screening leads to non-metal chelating metallo-β-lactamase inhibitors. Chem. Sci..

[27] Varlamov A.V., Boltukhina E.V., Zubkov F.I., Sidorenko N.V., Chernyshev A.I., Grudinin A.I. (2004). Preparative synthesis of 7-carboxy-2-R-isoindol-1-ones. Chem. Heterocycl. Compd..

[28] (2008). SAINT and SADABS.

[29] Steyermark A. (1961). Quantitative Organic Microanalysis.

[30] Neese F. (2022). Software update: The ORCA program system—Version 5.0 Wiley Interdiscip. Rev. Comput. Mol. Sci..

[31] Chemcraft—Graphical Software for Visualization of Quantum Chemistry Computations. https://www.chemcraftprog.com.

[32] Avogadro—An Advanced Molecule Editor and Visualizer. https://avogadro.cc/.

[33] Grimme S., Antony J., Ehrlich S., Krieg H. (2010). A consistent and accurate ab initio parametrization of density functionaldispersion correction (DFT-D) for the 94 elements H-Pu. J. Chem. Phys..

[34] Grimme S., Hansen A., Brandenburg J.G., Bannwarth C. (2016). Dispersion-corrected mean-field electronic structure methods. Chem. Rev..

[35] Goerigk L., Hansen A., Bauer C., Ehrlich S., Najibi A., Grimme S. (2017). A look at the density functional theory zoo withthe advanced GMTKN55 database for general main group thermochemistry, kinetics and noncovalent interactions. Phys. Chem. Chem. Phys..

[36] Raju R.K., Bengali A.A., Brothers E.N. (2016). A unified set of experimental organometallic data used to evaluate modern theoretical methods. Dalton Trans..

[37] Becke A.D. (1993). Density-functional thermochemistry. III. The role of exact exchange. J. Chem. Phys..

[38] Lee C., Yang W., Parr R.G. (1988). Development of the Colle-Salvetti correlation-energy formula into a functional of the electron density. Phys. Rev. B.

[39] Makrlik E., Toman P., Vanura P. (2012). A combined experimental and DFT study on the complexation of Mg^2+^ with beauvericin E. Struct. Chem..

[40] Fang J.L., Li W.Z., Zhou J., Chen J., Xia C. (2011). Novel guanidinium zwitterion and derived ionic liquids: Physicochemical properties and DFT theoretical studies. Struct. Chem..

[41] Dunning T.H. (1989). Gaussian basis sets for use in correlated molecular calculations. I. The atoms boron through neon and hydrogen. J. Chem. Phys..

[42] Hay P.J., Wadt W.R. (1985). Ab Initio Effective Core Potentials for Molecular Calculations. Potentials for the Transition Metal Atoms Sc to Hg. J. Chem. Phys..

[43] Hay P.J., Wadt W.R. (1985). Ab initio effective core potentials for molecular calculations. Potentials for K to Au including the outermost core orbitals. J. Chem. Phys..

[44] Wadt W.R., Hay P.J. (1985). Ab Initio Effective Core Potentials for Molecular Calculations. Potentials for main group elements Na to Bi. J. Chem. Phys..

[45] Tomasi J., Mennucci B., Cammi R. (2005). Quantum mechanical continuum solvation models. Chem. Rev..

[46] Krause L., Herbst-Irmer R., Sheldrick G.M., Stalke D. (2015). Comparison of silver and molybdenum microfocus X-ray sources for single-crystal structure determination. J. Appl. Cryst..

[47] Sheldrick  G.M. (2015). SHELXT—Integrated space-group and crystal-structure determination. Acta Crystallogr..

[48] Sheldrick G.M. (2015). Crystal structure refinement with SHELXL. Acta Crystallogr..

