# Supplementary material for: Synthesis, Structure, and Antitumor Activity of Heterocyclic 2-[4-(Dimethylamino)benzyl]-3-oxoisoindoline-4-carboxylates
Source: Molecules. 2026 May 5;31(9):1528. doi: 10.3390/molecules31091528 (PMC13165306; doi:10.3390/molecules31091528)
Supplement: Supplementary file 1 [file molecules-31-01528-s001.zip › molecules-4266559-supplementary.pdf]

Electronic supplementary materials for the paper

Synthesis, Structure, and Antitumor Activity of Heterocyclic 2-[4-(Dimethylamino)benzyl]-3-oxoisoindoline-4-carboxylates

Gulim K. Mukusheva [1], Roza I. Jalmakhanbetova [2], Zharkyn Zh. Zhumagaliyeva [3], Gulzhaukhar A. Toktarbay [1], Irina A. Kolesnik [4,*], Ekaterina A. Akishina [4], Evgenij A. Dikusar [4], Vladimir I. Potkin [4], Aliaksandr L. Pushkarchuk [5], Tatiana I. Terpinskaya [6], Fedor I. Zubkov [7], Mikhail S. Grigoriev [8] and Hongwei Zhou [9]

[1] Chemistry Faculty, NLC "Karaganda National Research University Named After Academician Ye.A. Buketov", Karaganda 100024, Kazakhstan; mukushevagulim5@gmail.com (G.K.M.); gulzhaukhar2001@gmail.com (G.A.T.)
[2] Department of Chemistry, Institute of Natural Sciences, L.N. Gumilyov Eurasian National University, Astana 010000, Kazakhstan; rjalmakhanbetova@gmail.com
[3] Astana International University, Astana 010000, Kazakhstan; zharkyn.73@mail.ru
[4] Laboratory of Chemistry of Heterocyclic Compounds, Institute of Physical Organic Chemistry, National Academy of Sciences of Belarus, 220072 Minsk, Belarus; che.semenovaea@mail.ru (E.A.A.); evgen_58@mail.ru (E.A.D.); potkin@ifoch.bas-net.by (V.I.P.)
[5] Laboratory of Ion Exchange and Sorption, Institute of Physical Organic Chemistry, National Academy of Sciences of Belarus, 220072 Minsk, Belarus; alexp51@bk.ru
[6] Center of Medical Microbiology and Antibiotic Resistance, Institute of Physiology, National Academy of Sciences of Belarus, 220072 Minsk, Belarus; terpinskayat@mail.ru
[7] Faculty of Science, Peoples Friendship University of Russia (RUDN University), Moscow 117198, Russia; fzubkov1973@gmail.com
[8] Frumkin Institute of Physical Chemistry and Electrochemistry, Russian Academy of Sciences, Moscow 119071, Russia; mickgrig@mail.ru
[9] College of Chemistry, Biology and Chemical Engineering, Jiaxing University, Jiaxing 314001, China; zhouhw@zju.edu.cn
* Correspondence: irynakolesnik93@gmail.com; Tel.: +375-(17)3971600

Table of contents

1. XRD part S2

2. Anticancer activity testing results S10

1. XRD part

X-ray diffraction experiments were carried out on an automatic four-circle area-detector diffractometer Bruker KAPPA APEX II (MoKα radiation). The unit cell constants were refined over the whole data set together with data reduction (SAINT-Plus (Version 8.40B), Bruker AXS Inc., Madison, Wisconsin, USA). The experimental intensities were corrected for absorption using the SADABS program [1]. The structures were solved by the intrinsic phasing method (SHELXT [2]) and refined by the full-matrix least-squares method (SHELXL-2018/3 [3]) on F^2 for all data in the anisotropic approximation for all non-hydrogen atoms. The H atoms of CH, CH_2 and CH_3 groups were placed in geometrically calculated positions with $U_{iso}(H) = 1.2U_{equ}(C)$ for CH and CH_2 groups and $U_{iso}(H) = 1.5U_{equ}(C)$ for CH_3 ones. The orientation of CH_3 groups was refined.

[1] Krause L.; Herbst-Irmer R.; Sheldrick G.M.; Stalke D. Comparison of silver and molybdenum microfocus X-ray sources for single-crystal structure determination. *J. Appl. Cryst*. **2015**, *48*, 3. Doi: 10.1107/S1600576714022985
[2] Sheldrick G.M. SHELXT – Integrated space-group and crystal-structure determination. *Acta Crystallogr*. **2015**, *A71*, 3–8. https://doi.org/10.1107/S2053273314026370
[3] Sheldrick G.M. Crystal structure refinement with SHELXL. *Acta Crystallogr*. **2015**, *C71*, 3–8. http://dx.doi.org/10.1107/S2053229614024218

Table S1. Crystal data and structure refinement for compounds **3a** and **3b.**

**Identification code**	**3a**	**3b**
CCDC deposition number	2488006	2488007
Empirical formula	$C_{28}H_{25}N_3O_4$	$C_{29}H_{27}N_3O_4$
Formula weight	467.51	481.53
Temperature/K	100(2)	100(2)
Crystal system	monoclinic	monoclinic
Space group	$P2_1/n$	$P2_1/n$
a/Å	13.658(5)	13.699(3)
b/Å	8.204(3)	8.1243(14)
c/Å	21.533(8)	22.140(4)
α/°	90	90
β/°	102.811(15)	105.446(5)
γ/°	90	90
Volume/Å^3	2352.7(15)	2375.1(8)
Z	4	4
ρ_{calc}g/cm^3	1.320	1.347
μ/mm^{-1}	0.089	0.091
F(000)	984.0	1016.0
Crystal size/mm^3	0.22 × 0.06 × 0.02	0.26 × 0.12 × 0.03
Radiation	MoKα (λ = 0.71073)	MoKα (λ = 0.71073)
2Θ range for data collection/°	8.166 to 59.99	7.194 to 49.986
Index ranges	-19 ≤ h ≤ 19, -11 ≤ k ≤ 10, -30 ≤ l ≤ 30	-16 ≤ h ≤ 15, -9 ≤ k ≤ 9, -26 ≤ l ≤ 26
Reflections collected	37889	28444
Independent reflections	6834 [R_{int} = 0.3486, R_{sigma} = 0.3664]	4147 [R_{int} = 0.1410, R_{sigma} = 0.1164]
Data/restraints/parameters	6834/0/319	4147/0/328
Goodness-of-fit on F^2	0.911	0.991
Final R indexes [I>=2σ (I)]	R_1 = 0.0893, wR_2 = 0.1558	R_1 = 0.0579, wR_2 = 0.1200
Final R indexes [all data]	R_1 = 0.3284, wR_2 = 0.2421	R_1 = 0.1379, wR_2 = 0.1536
Largest diff. peak/hole/e Å^{-3}	0.31/-0.28	0.27/-0.30

Table S2. Fractional Atomic Coordinates ($\times10^4$) and Equivalent Isotropic Displacement Parameters (Å$^2\times10^3$) for 2a and 2b. U_{eq} is defined as 1/3 of the trace of the orthogonalised U_{IJ} tensor.

**3a**					**3b**				
**Atom**	*x*	*y*	*z*	**U(eq)**	**Atom**	*x*	*y*	*z*	**U(eq)**
O1	5680(2)	5647(3)	6783.5(14)	37.6(8)	O1	3530.3(14)	6947(3)	4366.8(10)	24.7(6)
O2	7213(3)	4816(4)	7310.4(18)	63.7(12)	O2	2567.8(15)	5136(3)	2835.6(11)	30.2(6)
O3	6475(2)	6881(3)	5690.4(15)	43.3(9)	O3	4232.6(14)	5679(3)	3228.0(10)	23.8(6)
O11	3502(3)	9359(3)	6612.7(15)	45.8(9)	O11	6436.7(15)	9424(3)	3387.3(11)	30.6(6)
N2	6166(3)	5319(4)	4776.3(18)	36.5(10)	N1	-466.6(19)	6933(4)	5685.8(14)	31.8(8)
N3	10497(3)	6840(4)	4266(2)	46.6(11)	N2	3851.8(17)	5320(3)	5255.1(13)	21.5(7)
N12	4540(3)	9044(4)	6694(2)	47.3(12)	N12	5393.0(19)	9112(4)	3310.6(14)	31.8(8)
C1	6090(4)	3601(4)	4596(2)	37.4(12)	C1	3924(2)	3574(4)	5417.9(16)	23.1(8)
C3	6312(3)	5551(5)	5409(2)	35.5(12)	C3A	3702(2)	3968(4)	4337.3(16)	18.5(8)
C3A	6269(3)	3914(4)	5705(2)	30.8(11)	C3	3681(2)	5598(4)	4629.7(16)	20.2(8)
C4	6397(3)	3466(5)	6337(2)	32.4(11)	C4	3539(2)	3550(4)	3706.8(16)	20.1(8)
C5	6445(3)	1806(5)	6489(2)	38.6(12)	C5	3509(2)	1902(4)	3547.8(16)	23.4(8)
C6	6337(4)	654(5)	6001(3)	48.4(14)	C6	3651(2)	691(4)	4009.4(16)	24.6(8)
C7	6182(4)	1102(5)	5370(3)	42.8(13)	C7A	3824(2)	2764(4)	4796.6(15)	19.5(8)
C7A	6169(3)	2762(5)	5223(2)	34.2(12)	C7	3813(2)	1117(4)	4639.4(17)	24.6(8)
C8	6506(4)	4701(5)	6865(2)	38.7(12)	C8	3718(2)	6606(4)	5694.0(16)	24.9(8)
C9	5737(4)	7025(5)	7221(2)	41.8(13)	C9	3367(2)	4859(4)	3213.2(16)	21.9(8)
C10	6337(4)	6604(5)	4337(2)	41.8(13)	C10	4136(2)	7104(4)	2814.3(16)	26.9(9)
C13	4708(4)	7739(5)	7071(2)	37.9(12)	C13	5182(2)	7815(4)	2951.1(16)	26.3(8)
C14	3826(4)	7214(5)	7241(2)	38.1(12)	C14	6037(2)	7232(4)	2772.4(16)	26.4(9)
C15	3090(4)	8235(4)	6951(2)	35.9(12)	C15	6805(2)	8263(4)	3052.8(16)	24.3(8)
C21	2026(4)	8354(5)	6920(2)	37.4(12)	C21	7891(2)	8350(4)	3093.3(16)	23.9(8)
C22	1604(4)	7471(5)	7351(2)	41.1(13)	C22	8298(2)	7416(4)	2693.1(16)	27.9(9)
C23	586(4)	7533(5)	7326(2)	47.4(14)	C23	9324(2)	7439(4)	2746.1(16)	29.5(9)
C24	-39(4)	8463(5)	6861(2)	45.2(13)	C24	9982(2)	8389(4)	3201.3(17)	29.0(9)
C25	367(4)	9372(5)	6439(2)	45.0(13)	C25	9567(2)	9360(4)	3590.1(16)	28.4(9)
C26	1385(4)	9316(5)	6458(2)	43.2(13)	C26	8535(2)	9346(4)	3538.1(16)	26.1(8)
C31	7427(3)	6713(5)	4307(2)	33.9(12)	C27	11113(2)	8380(5)	3277.4(17)	37.1(10)
C32	7786(4)	5937(5)	3833(2)	39.3(12)	C31	2626(2)	6741(4)	5706.2(16)	22.9(8)
C33	8792(4)	5947(5)	3815(2)	39.1(12)	C32	1911(2)	7497(4)	5217.6(16)	26.2(9)
C34	9493(4)	6747(5)	4293(2)	36.1(12)	C33	894(2)	7537(4)	5203.4(16)	26.5(9)
C35	9138(4)	7492(5)	4781(2)	40.6(12)	C34	545(2)	6817(4)	5684.7(16)	24.2(8)
C36	8131(4)	7486(5)	4788(2)	40.4(13)	C35	1262(2)	6026(4)	6168.7(17)	26.9(9)
C37	11228(4)	7389(6)	4833(3)	61.7(16)	C36	2269(2)	6004(4)	6172.0(16)	23.7(8)
C38	10882(4)	5754(6)	3843(3)	59.4(16)	C37	-837(3)	5912(5)	6112.1(19)	44.9(11)
					C38	-1209(2)	7445(5)	5114.4(18)	40.2(11)

Table S3. Anisotropic Displacement Parameters (Å^2×10^3) for 2a and 2b. The Anisotropic displacement factor exponent takes the form: -2π^2[h^{2}a*^{2}U_{11}+2hka*b*U_{12}+…].

**3a**							**3b**						
**Atom**	**U_{11}**	**U_{22}**	**U_{33}**	**U_{23}**	**U_{13}**	**U_{12}**	**Atom**	**U_{11}**	**U_{22}**	**U_{33}**	**U_{23}**	**U_{13}**	**U_{12}**
O1	42(2)	27.6(16)	41(2)	-7.8(13)	6.3(17)	-0.4(13)	O1	28.8(12)	18.4(14)	27.1(15)	1.1(12)	7.7(10)	0.5(10)
O2	62(3)	54(2)	61(3)	-18.4(18)	-17(2)	13.0(18)	O2	24.3(12)	34.7(16)	25.7(15)	6.6(12)	-3.5(11)	0.3(11)
O3	62(3)	20.4(16)	51(2)	-5.7(13)	18.9(19)	-1.0(13)	O3	23.7(12)	21.7(14)	25.1(15)	7.3(11)	5.0(10)	-1.9(10)
O11	52(3)	27.4(17)	62(2)	9.0(15)	22(2)	4.9(14)	O11	27.8(13)	25.6(15)	40.9(17)	-3.8(12)	13.4(11)	-3.5(11)
N2	38(3)	27.2(19)	43(3)	1.2(16)	6(2)	0.5(15)	N1	21.0(15)	38(2)	36(2)	-0.7(16)	6.3(14)	-2.6(13)
N3	38(3)	49(2)	52(3)	4(2)	8(2)	1.7(19)	N2	19.5(14)	23.7(17)	21.3(18)	-3.1(14)	5.3(12)	0.3(12)
N12	49(3)	30(2)	67(3)	3.9(19)	23(3)	4.0(18)	N12	27.3(17)	30.6(19)	38(2)	-1.7(16)	9.6(14)	-3.6(13)
C1	38(3)	30(2)	43(3)	-5.3(19)	7(3)	-3.1(18)	C1	15.6(16)	25(2)	28(2)	5.6(16)	3.6(15)	0.0(14)
C3	27(3)	28(2)	50(3)	2(2)	5(2)	0.9(18)	C3A	13.4(16)	16.9(19)	26(2)	0.4(16)	6.9(14)	-2.7(13)
C3A	27(3)	20(2)	45(3)	-2.6(19)	5(2)	2.2(16)	C3	14.8(16)	22(2)	24(2)	-0.1(17)	5.9(14)	-0.3(14)
C4	28(3)	28(2)	40(3)	-4.5(19)	5(2)	1.5(17)	C4	12.5(16)	24(2)	23(2)	3.8(16)	3.3(14)	2.1(13)
C5	41(3)	28(2)	47(3)	5(2)	11(3)	-0.1(19)	C5	23.8(18)	23(2)	24(2)	-4.6(17)	7.2(15)	-2.5(15)
C6	59(4)	19(2)	67(4)	-4(2)	12(3)	-1(2)	C6	26.7(18)	15(2)	33(2)	0.8(17)	9.1(16)	-0.2(15)
C7	48(4)	20(2)	61(4)	-11(2)	13(3)	-3.1(19)	C7A	15.5(16)	22(2)	21(2)	0.6(16)	3.8(14)	-1.0(14)
C7A	31(3)	27(2)	46(3)	-3(2)	11(2)	-0.4(18)	C7	22.9(18)	21(2)	29(2)	7.5(17)	6.1(16)	1.4(15)
C8	47(4)	24(2)	45(3)	3(2)	7(3)	3(2)	C8	22.6(18)	29(2)	22(2)	-7.3(17)	3.2(15)	0.9(15)
C9	46(3)	30(2)	48(3)	-18(2)	8(3)	-4(2)	C9	23.7(18)	21(2)	23(2)	-1.9(16)	10.1(15)	-0.4(15)
C10	41(3)	34(2)	49(3)	14(2)	8(3)	4(2)	C10	29.6(19)	21(2)	27(2)	7.5(17)	2.6(16)	-0.3(16)
C13	44(4)	27(2)	44(3)	-8(2)	12(3)	-1(2)	C13	30.7(19)	23(2)	24(2)	6.7(17)	5.7(16)	0.1(16)
C14	47(4)	28(2)	38(3)	1.3(19)	6(3)	-1(2)	C14	31.5(19)	22(2)	24(2)	-3.8(17)	5.3(16)	0.9(16)
C15	54(4)	18(2)	36(3)	-4.9(18)	13(3)	0(2)	C15	30.3(19)	18(2)	24(2)	7.2(17)	6.3(16)	5.5(15)
C21	47(4)	23(2)	42(3)	-3(2)	9(3)	0.7(19)	C21	28.0(19)	20(2)	23(2)	1.5(16)	5.7(15)	0.1(15)
C22	52(4)	26(2)	46(3)	0(2)	12(3)	-1(2)	C22	34(2)	24(2)	24(2)	-3.9(17)	5.3(16)	0.4(16)
C23	52(4)	42(3)	48(4)	-2(2)	12(3)	-2(2)	C23	34(2)	27(2)	30(2)	2.9(17)	13.5(17)	4.3(16)
C24	44(4)	38(3)	53(4)	-7(2)	10(3)	0(2)	C24	31(2)	26(2)	29(2)	10.4(18)	8.0(17)	3.7(16)
C25	50(4)	37(3)	46(3)	-3(2)	6(3)	2(2)	C25	32(2)	25(2)	25(2)	0.9(17)	1.8(16)	-1.6(16)
C26	59(4)	28(2)	41(3)	-2(2)	6(3)	-1(2)	C26	33(2)	22(2)	23(2)	2.3(17)	7.6(16)	0.3(16)
C31	38(3)	25(2)	38(3)	4.1(19)	6(3)	0.7(18)	C27	33(2)	40(3)	38(3)	8(2)	9.4(18)	3.8(17)
C32	47(4)	30(2)	40(3)	1(2)	7(3)	-4(2)	C31	20.9(17)	22(2)	25(2)	-1.6(17)	5.2(15)	-2.4(15)
C33	50(4)	33(2)	37(3)	-6(2)	15(3)	-2(2)	C32	28.4(19)	28(2)	24(2)	-2.0(17)	9.8(16)	1.8(16)
C34	32(3)	32(2)	44(3)	7(2)	9(3)	4.7(19)	C33	25.0(18)	30(2)	21(2)	2.0(17)	1.6(15)	3.5(15)
C35	40(4)	38(3)	42(3)	-1(2)	3(3)	-6(2)	C34	20.0(18)	21(2)	31(2)	-6.3(17)	7.2(15)	-1.2(14)
C36	48(4)	31(2)	41(3)	-4(2)	7(3)	0(2)	C35	32(2)	23(2)	30(2)	2.1(17)	14.7(17)	1.0(15)
C37	40(4)	72(4)	68(4)	9(3)	-2(3)	-4(3)	C36	25.4(19)	22(2)	21(2)	-1.4(16)	1.9(15)	2.9(15)
C38	44(4)	72(4)	68(4)	11(3)	26(3)	7(3)	C37	27(2)	63(3)	48(3)	-3(2)	13.7(19)	-9.7(19)
							C38	21.1(18)	55(3)	42(3)	-3(2)	4.3(17)	0.8(18)

Table S4. Bond Lengths for **3a** and **3b**.

3a				3b		
**Atom**	**Atom**	**Length/Å**		**Atom**	**Atom**	**Length/Å**
O1	C8	1.348(5)		O1	C3	1.232(4)
O1	C9	1.463(4)		O2	C9	1.209(3)
O2	C8	1.205(5)		O3	C9	1.353(3)
O3	C3	1.244(5)		O3	C10	1.460(4)
O11	N12	1.413(5)		O11	N12	1.417(3)
O11	C15	1.371(5)		O11	C15	1.375(4)
N2	C1	1.460(5)		N1	C34	1.389(4)
N2	C3	1.345(5)		N1	C37	1.446(4)
N2	C10	1.469(5)		N1	C38	1.457(4)
N3	C34	1.388(6)		N2	C1	1.460(4)
N3	C37	1.467(6)		N2	C3	1.360(4)
N3	C38	1.453(6)		N2	C8	1.471(4)
N12	C13	1.333(5)		N12	C13	1.305(4)
C1	C7A	1.499(6)		C1	C7A	1.498(4)
C3	C3A	1.494(5)		C3A	C3	1.477(4)
C3A	C4	1.382(6)		C3A	C4	1.396(4)
C3A	C7A	1.388(6)		C3A	C7A	1.388(4)
C4	C5	1.398(5)		C4	C5	1.382(4)
C4	C8	1.506(6)		C4	C9	1.498(4)
C5	C6	1.396(6)		C5	C6	1.394(4)
C6	C7	1.377(6)		C6	C7	1.396(4)
C7	C7A	1.397(5)		C7A	C7	1.382(4)
C9	C13	1.491(6)		C8	C31	1.507(4)
C10	C31	1.507(6)		C10	C13	1.499(4)
C13	C14	1.403(6)		C13	C14	1.414(4)
C14	C15	1.350(6)		C14	C15	1.358(4)
C15	C21	1.443(6)		C15	C21	1.468(4)
C21	C22	1.399(6)		C21	C22	1.390(4)
C21	C26	1.412(6)		C21	C26	1.393(4)
C22	C23	1.380(6)		C22	C23	1.380(4)
C23	C24	1.391(6)		C23	C24	1.393(5)
C24	C25	1.384(6)		C24	C25	1.396(5)
C25	C26	1.383(6)		C24	C27	1.513(4)
C31	C32	1.383(6)		C25	C26	1.387(4)
C31	C36	1.399(6)		C31	C32	1.393(4)
C32	C33	1.382(6)		C31	C36	1.389(4)
C33	C34	1.403(6)		C32	C33	1.387(4)
C34	C35	1.392(6)		C33	C34	1.407(4)
C35	C36	1.379(6)		C34	C35	1.402(4)
				C35	C36	1.379(4)

Table S5. Bond Angles for **3a** and **3b**.

3a					3b			
**Atom**	**Atom**	**Atom**	**Angle/°**		**Atom**	**Atom**	**Atom**	**Angle/°**
C8	O1	C9	115.9(3)		C9	O3	C10	116.3(2)
C15	O11	N12	109.3(3)		C15	O11	N12	108.4(2)
C1	N2	C10	122.2(4)		C34	N1	C37	119.0(3)
C3	N2	C1	113.1(3)		C34	N1	C38	119.0(3)
C3	N2	C10	122.8(3)		C37	N1	C38	116.4(3)
C34	N3	C37	118.5(4)		C1	N2	C8	122.6(3)
C34	N3	C38	119.4(4)		C3	N2	C1	113.2(3)
C38	N3	C37	115.9(4)		C3	N2	C8	122.4(3)
C13	N12	O11	104.7(3)		C13	N12	O11	105.7(3)
N2	C1	C7A	102.5(3)		N2	C1	C7A	102.5(3)
O3	C3	N2	126.0(4)		C4	C3A	C3	129.8(3)
O3	C3	C3A	126.8(4)		C7A	C3A	C3	108.8(3)
N2	C3	C3A	107.2(3)		C7A	C3A	C4	121.1(3)
C4	C3A	C3	130.7(4)		O1	C3	N2	126.2(3)
C4	C3A	C7A	121.6(4)		O1	C3	C3A	127.6(3)
C7A	C3A	C3	107.4(4)		N2	C3	C3A	106.2(3)
C3A	C4	C5	118.6(4)		C3A	C4	C9	120.6(3)
C3A	C4	C8	122.3(4)		C5	C4	C3A	118.4(3)
C5	C4	C8	119.2(4)		C5	C4	C9	120.9(3)
C6	C5	C4	119.5(4)		C4	C5	C6	120.5(3)
C7	C6	C5	121.9(4)		C5	C6	C7	120.8(3)
C6	C7	C7A	118.3(4)		C3A	C7A	C1	109.1(3)
C3A	C7A	C1	109.7(4)		C7	C7A	C1	130.5(3)
C3A	C7A	C7	120.0(4)		C7	C7A	C3A	120.4(3)
C7	C7A	C1	130.3(4)		C7A	C7	C6	118.7(3)
O1	C8	C4	110.4(4)		N2	C8	C31	111.3(2)
O2	C8	O1	124.0(4)		O2	C9	O3	123.5(3)
O2	C8	C4	125.5(4)		O2	C9	C4	125.1(3)
O1	C9	C13	104.5(3)		O3	C9	C4	111.4(3)
N2	C10	C31	111.7(3)		O3	C10	C13	104.9(2)
N12	C13	C9	118.3(4)		N12	C13	C10	118.6(3)
N12	C13	C14	111.3(4)		N12	C13	C14	112.0(3)
C14	C13	C9	130.3(4)		C14	C13	C10	129.3(3)
C15	C14	C13	106.5(4)		C15	C14	C13	105.3(3)
O11	C15	C21	117.3(4)		O11	C15	C21	116.4(3)
C14	C15	O11	108.1(4)		C14	C15	O11	108.6(3)
C14	C15	C21	134.5(4)		C14	C15	C21	134.9(3)
C22	C21	C15	119.6(4)		C22	C21	C15	120.4(3)
C22	C21	C26	118.4(5)		C22	C21	C26	118.8(3)
C26	C21	C15	122.0(4)		C26	C21	C15	120.8(3)
C23	C22	C21	121.0(5)		C23	C22	C21	120.7(3)
C22	C23	C24	120.1(5)		C22	C23	C24	121.1(3)
C25	C24	C23	119.8(5)		C23	C24	C25	117.9(3)
C26	C25	C24	120.6(5)		C23	C24	C27	121.6(3)
C25	C26	C21	120.1(5)		C25	C24	C27	120.5(3)
C32	C31	C10	121.3(4)		C26	C25	C24	121.2(3)
C32	C31	C36	117.2(5)		C25	C26	C21	120.2(3)
C36	C31	C10	121.2(4)		C32	C31	C8	120.9(3)
C33	C32	C31	122.3(4)		C36	C31	C8	121.9(3)
C32	C33	C34	120.2(4)		C36	C31	C32	116.9(3)
N3	C34	C33	121.0(5)		C33	C32	C31	121.5(3)
N3	C34	C35	121.4(4)		C32	C33	C34	121.0(3)
C35	C34	C33	117.6(5)		N1	C34	C33	120.9(3)
C36	C35	C34	121.5(5)		N1	C34	C35	121.7(3)
C35	C36	C31	121.1(5)		C35	C34	C33	117.3(3)
					C36	C35	C34	120.4(3)
					C35	C36	C31	122.7(3)

Table S6. Torsion Angles for **3a** and **3b**.

3a										3b									
**A**	**B**	**C**	**D**	**Angle/°**	**A**	**B**	**C**	**D**	**Angle/°**	**A**	**B**	**C**	**D**	**Angle/°**	**A**	**B**	**C**	**D**	**Angle/°**
O1	C9	C13	N12	-98.9(4)	C6	C7	C7A	C3A	-2.7(7)	O3	C10	C13	N12	-101.3(3)	C7A	C3A	C4	C5	-0.4(4)
O1	C9	C13	C14	77.6(6)	C7A	C3A	C4	C5	1.2(7)	O3	C10	C13	C14	74.8(4)	C7A	C3A	C4	C9	177.6(3)
O3	C3	C3A	C4	-0.5(8)	C7A	C3A	C4	C8	-180.0(4)	O11	N12	C13	C10	176.2(3)	C8	N2	C1	C7A	168.2(2)
O3	C3	C3A	C7A	-174.2(4)	C8	O1	C9	C13	-174.3(4)	O11	N12	C13	C14	-0.6(4)	C8	N2	C3	O1	10.0(4)
O11	N12	C13	C9	176.6(4)	C8	C4	C5	C6	179.5(4)	O11	C15	C21	C22	-169.2(3)	C8	N2	C3	C3A	-169.4(2)
O11	N12	C13	C14	-0.6(5)	C9	O1	C8	O2	9.0(7)	O11	C15	C21	C26	11.8(5)	C8	C31	C32	C33	175.8(3)
O11	C15	C21	C22	-166.9(4)	C9	O1	C8	C4	-172.9(3)	N1	C34	C35	C36	-176.6(3)	C8	C31	C36	C35	-175.7(3)
O11	C15	C21	C26	14.3(6)	C9	C13	C14	C15	-176.3(4)	N2	C1	C7A	C3A	-0.3(3)	C9	O3	C10	C13	177.0(3)
N2	C1	C7A	C3A	0.6(5)	C10	N2	C1	C7A	166.9(4)	N2	C1	C7A	C7	-178.5(3)	C9	C4	C5	C6	-178.8(3)
N2	C1	C7A	C7	-177.6(4)	C10	N2	C3	O3	10.0(7)	N2	C8	C31	C32	-75.5(4)	C10	O3	C9	O2	8.3(4)
N2	C3	C3A	C4	178.1(5)	C10	N2	C3	C3A	-168.6(4)	N2	C8	C31	C36	98.9(4)	C10	O3	C9	C4	-173.4(3)
N2	C3	C3A	C7A	4.4(5)	C10	C31	C32	C33	-176.5(4)	N12	O11	C15	C14	-0.3(3)	C10	C13	C14	C15	-175.9(3)
N2	C10	C31	C32	96.3(5)	C10	C31	C36	C35	175.8(4)	N12	O11	C15	C21	-177.9(3)	C13	C14	C15	O11	-0.1(4)
N2	C10	C31	C36	-78.2(5)	C13	C14	C15	O11	-0.1(5)	N12	C13	C14	C15	0.4(4)	C13	C14	C15	C21	176.9(4)
N3	C34	C35	C36	176.0(4)	C13	C14	C15	C21	178.8(5)	C1	N2	C3	O1	175.4(3)	C14	C15	C21	C22	14.0(6)
N12	O11	C15	C14	-0.3(5)	C14	C15	C21	C22	14.3(8)	C1	N2	C3	C3A	-3.9(3)	C14	C15	C21	C26	-165.0(4)
N12	O11	C15	C21	-179.4(4)	C14	C15	C21	C26	-164.5(5)	C1	N2	C8	C31	-80.7(3)	C15	O11	N12	C13	0.5(3)
N12	C13	C14	C15	0.4(5)	C15	O11	N12	C13	0.6(5)	C1	C7A	C7	C6	176.4(3)	C15	C21	C22	C23	-177.2(3)
C1	N2	C3	O3	174.4(4)	C15	C21	C22	C23	-178.7(4)	C3A	C4	C5	C6	-0.8(4)	C15	C21	C26	C25	177.1(3)
C1	N2	C3	C3A	-4.2(5)	C15	C21	C26	C25	178.8(4)	C3A	C4	C9	O2	-108.9(4)	C21	C22	C23	C24	0.2(5)
C1	N2	C10	C31	-79.8(5)	C21	C22	C23	C24	1.0(7)	C3A	C4	C9	O3	72.9(3)	C22	C21	C26	C25	-1.9(5)
C3	N2	C1	C7A	2.4(5)	C22	C21	C26	C25	0.1(6)	C3A	C7A	C7	C6	-1.5(4)	C22	C23	C24	C25	-2.0(5)
C3	N2	C10	C31	83.2(5)	C22	C23	C24	C25	-2.3(7)	C3	N2	C1	C7A	2.7(3)	C22	C23	C24	C27	178.0(3)
C3	C3A	C4	C5	-171.7(4)	C23	C24	C25	C26	2.6(7)	C3	N2	C8	C31	83.4(4)	C23	C24	C25	C26	1.9(5)
C3	C3A	C4	C8	7.1(7)	C24	C25	C26	C21	-1.4(7)	C3	C3A	C4	C5	-174.1(3)	C24	C25	C26	C21	0.1(5)
C3	C3A	C7A	C1	-3.0(5)	C26	C21	C22	C23	0.1(6)	C3	C3A	C4	C9	3.9(5)	C26	C21	C22	C23	1.8(5)
C3	C3A	C7A	C7	175.4(4)	C31	C32	C33	C34	0.7(6)	C3	C3A	C7A	C1	-1.9(3)	C27	C24	C25	C26	-178.1(3)
C3A	C4	C5	C6	-1.7(7)	C32	C31	C36	C35	1.0(6)	C3	C3A	C7A	C7	176.5(3)	C31	C32	C33	C34	0.2(5)
C3A	C4	C8	O1	60.7(6)	C32	C33	C34	N3	-176.7(4)	C4	C3A	C3	O1	-1.5(5)	C32	C31	C36	C35	-1.1(5)
C3A	C4	C8	O2	-121.2(6)	C32	C33	C34	C35	1.0(6)	C4	C3A	C3	N2	177.8(3)	C32	C33	C34	N1	176.6(3)
C4	C3A	C7A	C1	-177.4(4)	C33	C34	C35	C36	-1.6(6)	C4	C3A	C7A	C1	-176.8(2)	C32	C33	C34	C35	-1.5(5)
C4	C3A	C7A	C7	1.0(7)	C34	C35	C36	C31	0.6(7)	C4	C3A	C7A	C7	1.6(4)	C33	C34	C35	C36	1.6(5)
C4	C5	C6	C7	0.0(7)	C36	C31	C32	C33	-1.7(6)	C4	C5	C6	C7	0.9(4)	C34	C35	C36	C31	-0.3(5)
C5	C4	C8	O1	-120.5(4)	C37	N3	C34	C33	-167.8(4)	C5	C4	C9	O2	69.1(4)	C36	C31	C32	C33	1.1(5)
C5	C4	C8	O2	57.6(7)	C37	N3	C34	C35	14.7(6)	C5	C4	C9	O3	-109.2(3)	C37	N1	C34	C33	167.7(3)
C5	C6	C7	C7A	2.2(7)	C38	N3	C34	C33	-16.5(6)	C5	C6	C7	C7A	0.3(4)	C37	N1	C34	C35	-14.2(5)
C6	C7	C7A	C1	175.3(5)	C38	N3	C34	C35	165.9(4)	C7A	C3A	C3	O1	-175.8(3)	C38	N1	C34	C33	15.0(5)
										C7A	C3A	C3	N2	3.5 (3)	C38	N1	C34	C35	-166.9(3)

Table S7. Hydrogen Atom Coordinates (Å×10[4]) and Isotropic Displacement Parameters (Å[2]×10[3]) for **3a** and **3b**.

**3a**						**3b**				
**Atom**	*x*	*y*	*z*	**U(eq)**		**Atom**	*x*	*y*	*z*	**U(eq)**
H1A	6644.16	3274.55	4393.72	45		H1A	3371.37	3235.1	5601.94	28
H1B	5440.1	3361.8	4300.92	45		H1B	4584.05	3313.23	5716.24	28
H5A	6551.25	1465.36	6919.99	46		H5A	3389.93	1591.91	3120.63	28
H6A	6370.7	-471.82	6107.32	58		H6A	3638.01	-436.35	3893.92	30
H7A	6085.74	302.2	5043.31	51		H7A	3912.69	290.64	4953.6	30
H9A	6239.43	7832.29	7148.91	50		H8A	3946.48	7676.42	5567.03	30
H9B	5921.64	6653.89	7669.43	50		H8B	4141.9	6344.71	6119.9	30
H10A	6117.27	7664.32	4478.28	50		H10A	3654.75	7915.56	2905.06	32
H10B	5926.74	6374.87	3905.8	50		H10B	3895.52	6766.61	2369.65	32
H14A	3758.53	6316.44	7506.9	46		H14A	6067.92	6315.71	2511.74	32
H22A	2024.31	6819.58	7666.3	49		H22A	7863.84	6756.06	2379.83	33
H23A	312.39	6939.93	7626.8	57		H23A	9587.06	6795.41	2467.23	35
H24A	-742.98	8473.63	6834.03	54		H25A	9998.19	10043.73	3895.71	34
H25A	-57.75	10041.36	6132.9	54		H26A	8266.73	10016.81	3806.78	31
H26A	1654.19	9925.8	6159.54	52		H27A	11389.99	9474.73	3408	56
H32A	7325.81	5377.68	3506.93	47		H27B	11246.83	8090.24	2877.38	56
H33A	9009.02	5410.62	3478.74	47		H27C	11433.78	7569.05	3596.28	56
H35A	9599.19	8017.7	5115.77	49		H32A	2126.45	7995.67	4886.75	31
H36A	7911.06	8014.82	5125.5	49		H33A	424.74	8059.78	4863.16	32
H37A	11898.46	7423.4	4740.69	93		H35A	1051.71	5500.97	6496.54	32
H37B	11229.27	6630.54	5184.89	93		H36A	2739.35	5463.73	6506.79	28
H37C	11043.77	8480.67	4953.11	93		H37A	-1547.95	6180.38	6076.1	67
H38A	11585.86	6020.97	3855.99	89		H37B	-433.33	6114.38	6542.6	67
H38B	10485.88	5885.86	3406.58	89		H37C	-781.41	4750.91	6006.64	67
H38C	10833.87	4623.84	3979.76	89		H38A	-1873.82	7561.52	5197.47	60
						H38B	-1249.35	6614.14	4787.63	60
						H38C	-1004.8	8502.13	4973.63	60

Figure S1. Packing diagram of **3a** and **3b**. Dotted lines show π-π interactions.

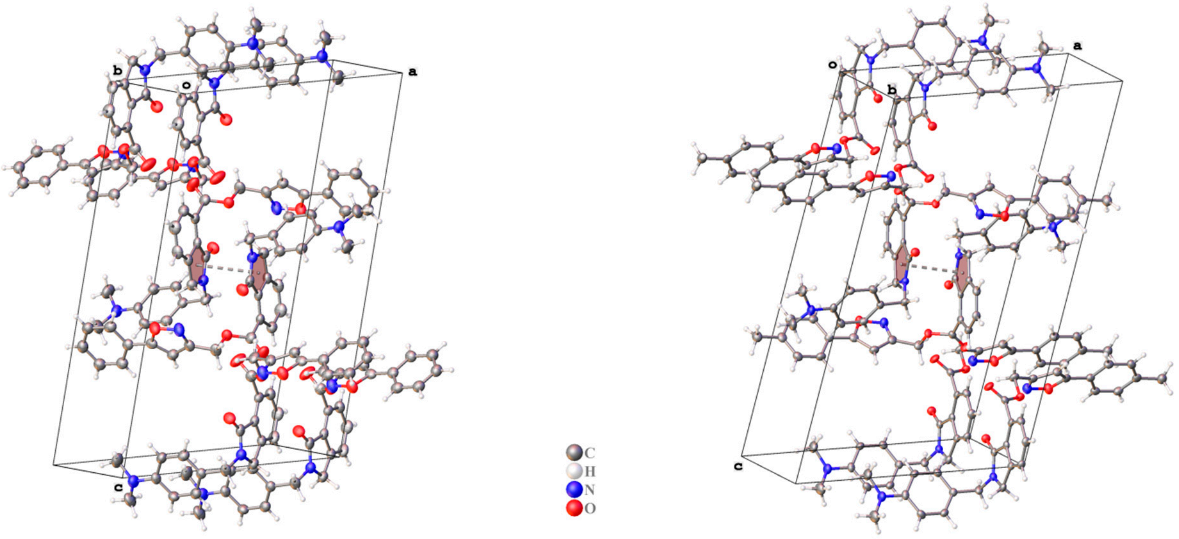

3a **3b**

2. Anticancer activity testing results

Cell lines. The research was carried out on Hela (cervical cancer, human) and C6 glioma (rat) cell lines from the collection of the The Republican Research and Practical Center for Epidemiology and Microbiology (RRPCEM, Republic of Belarus).

Sample preparation. The solutions (0.5 mM stock) of the test samples were prepared in 0.9% isotonic sodium chloride solution. Further dilutions were made in isotonic sodium chloride solution. The solutions were added to the wells of the plate with growing cells in a volume ratio of 1 (test compounds) : 9 (medium with cells).

Antitumor drugs: carboplatin (*Fresenius Kabi*, Germany), doxorubicin (*RUPE "Belmedpreparaty"*, Belarus), cyclophosphamide (*RUPE "Belmedpreparaty"*, Belarus), fluorouracil (*RUPE "Belmedpreparaty"*, Belarus).

Conducting experiments. The cells were seeded into wells of 96-well plates (Corning) in Dulbecco's modified eagle medium 5648 (“*Sigma-Aldrich*”, USA) supplemented with 10% fetal bovine serum (“*Sigma-Aldrich*”, USA) and antibiotics (penicillin, streptomycin, amphotericin B, “*Biological Industries*”, USA). After 24 h, the test compounds at a final concentration of 100 or 200 μM and/or antitumor drugs at a final concentration of 5–50 μM were added to the wells. Isotonic sodium chloride solution was added as a control. The cells were cultured for 48 h at 37 °C and 5% CO_2. Then, the cell samples were analyzed using the standard MTT tests with 3-(4,5-dimethylthiazol-2-yl)-2,5-diphenyl-tetrazolium bromide (“Glentham Life Sciences”, UK).

Statistical processing. The resulting digital material was processed by methods of variation statistics using the Excel and Statistica 7 software packages. The data are presented as the mean and its standard error. Differences between series were considered significant at a significance level of $p < 0.05$ according to Student's t test.

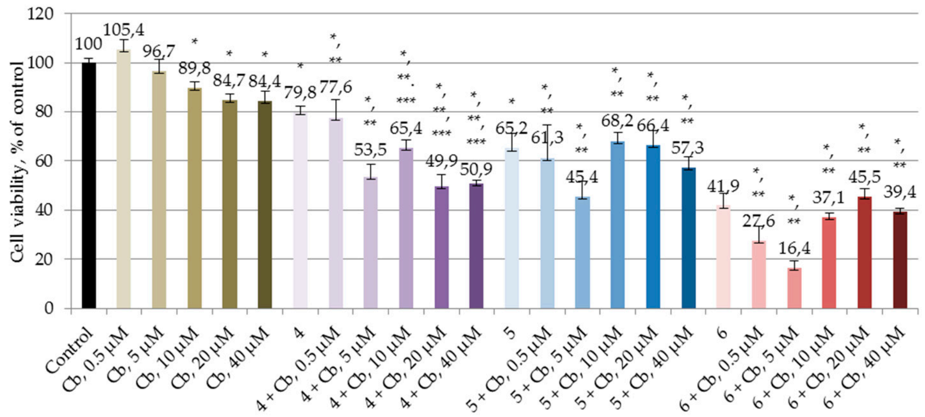

Figure S2. Effect of **5**–**7** (200 µM), carboplatin (0.5–40 µM) and their mixtures on the viability of HeLa cells, *$p < 0.05$ when compared with the control; **$p < 0.05$ when compared with the effect of carboplatin at the corresponding dose (for series where the combined action of drugs was used); ***$p < 0.05$ when compared with the effect of the corresponding heterocyclic drug (for series where the combined action of drugs was used)

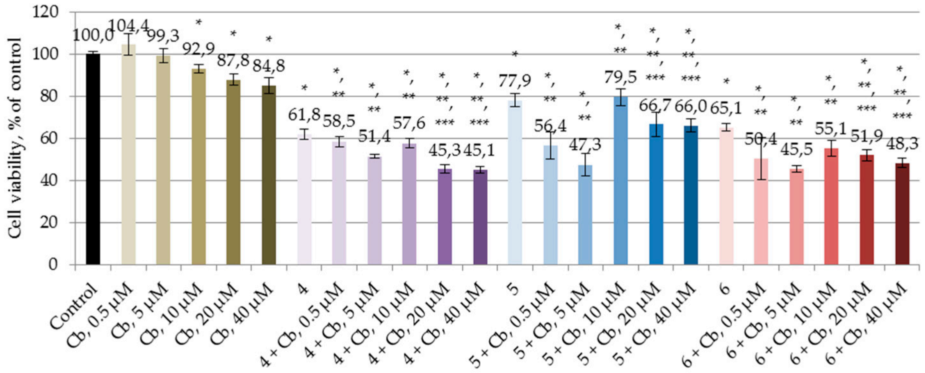

Figure S3. Effect of **5**–**7** (200 µM), carboplatin (0.5–40 µM) and their mixtures on the viability of glioma C6 cells, *$p < 0.05$ when compared with the control; **$p < 0.05$ when compared with the effect of carboplatin at the corresponding dose (for series where the combined action of drugs was used); ***$p < 0.05$ when compared with the effect of the corresponding heterocyclic drug (for series where the combined action of drugs was used)

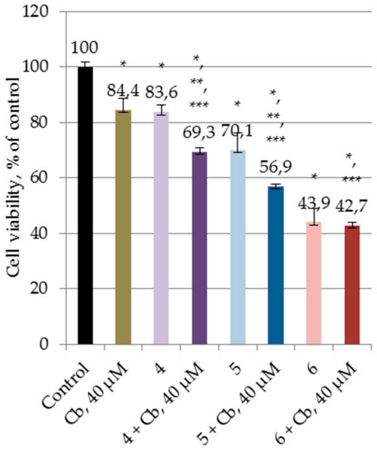

(a)

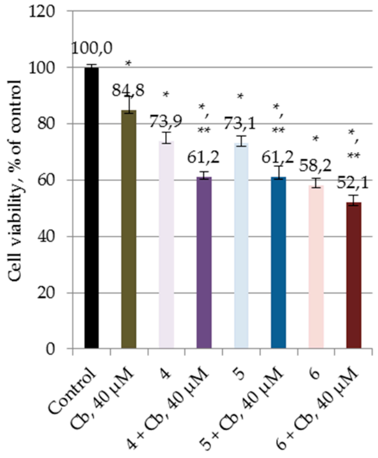

(b)

Figure S4. Effect of **5**–**7** (100 µM), carboplatin (40 µM) and their mixtures on the viability of Hela (a) and glioma C6 (b) cells, *$p < 0.05$ when compared with the control; **$p < 0.05$ when compared with the effect of the corresponding heterocyclic drug (for series where the combined action of drugs was used); ***$p < 0.05$ when compared with the effect of carboplatin at the corresponding dose (for series where the combined action of drugs was used)

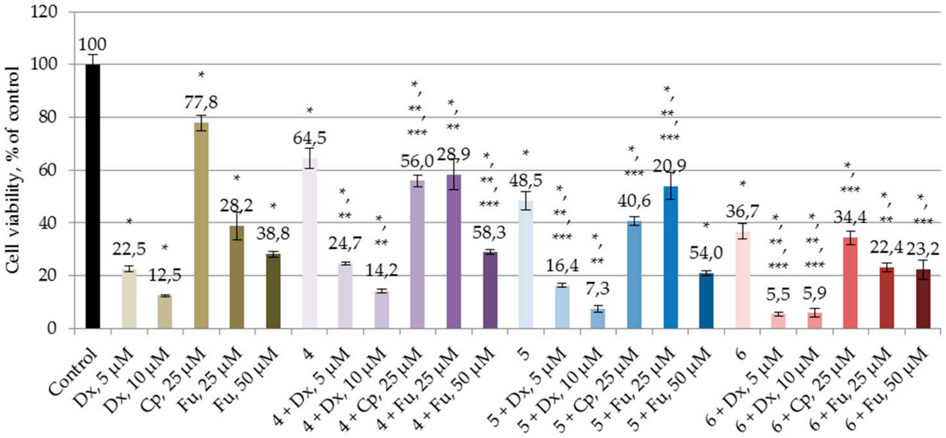

Figure S5. Effect of **5**–**7** (200 µM) in compositions with doxorubicin (5 or 10 µM), cyclophosphamide (25 µM) or fluorouracil (25 or 50 µM) on the viability of Hela cells, *p < 0.05 when compared with the control; **p < 0.05 when compared with the effect of the corresponding heterocyclic drug (for series where the combined action of drugs was used); ***p < 0.05 when compared with the effect of carboplatin at the corresponding dose (for series where the combined action of drugs was used)

Table S8. Decrease in Hela cell viability due to the application of doxorubicin (5 or 10 µM), cyclophosphamide (25 µM) or fluorouracil (25 or 50 µM), compounds **5**–**7** and their compositions

Drug	Drug dose	Drug action, %	Compound tested (200 µM)	Joint action, %	Σ	Observed effect
-	0 µM	–	**5**	35.5	–	–
			**6**	51.5		
			**7**	63.3		
Doxorubicin	5 µM	77.5	**5**	75.3	113.0	Ant (~38%)
			**6**	83.6	129.0	Ant (~45%)
			**7**	94.5	140.8	Ant (~46%)
	10 µM	87.5	**5**	85.8	123.0	Ant (~37%)
			**6**	92.7	139.0	Ant (~46%)
			**7**	94.1	133.2	Ant (~39%)
Cyclophosphamide	25 µM	22.2	**5**	44.0	57.7	Ant (~14%)
			**6**	59.4	73.7	Ant (~14%)
			**7**	65.6	85.5	Ant (~20%)
Fluorouracil	25 µM	61.2	**5**	41.7	96.7	Ant (~55%)
			**6**	46.0	112.7	Ant (~67%)
			**7**	76.8	124.5	Ant (~48%)
	50 µM	71.8	**5**	71.1	107.3	Ant (~36%)
			**6**	79.1	123.3	Ant (~44%)
			**7**	77.6	135.1	Ant (~58%)

Σ – summarized effect of the drug and compound itself; Ant – antagonizm; Syn – synergism.

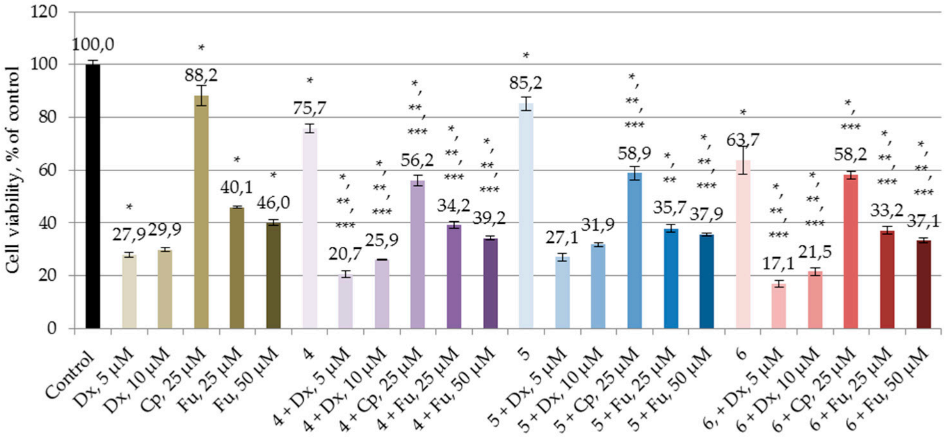

Figure S6. Effect of **5–7** (200 μM) in compositions with doxorubicin (5 or 10 μM), cyclophosphamide (25 μM) or fluorouracil (25 or 50 μM) on the viability of glioma C6 cells, *p < 0.05 when compared with the control; **p < 0.05 when compared with the effect of the corresponding heterocyclic drug (for series where the combined action of drugs was used); ***p < 0.05 when compared with the effect of carboplatin at the corresponding dose (for series where the combined action of drugs was used)

Table S9. Decrease in glioma C6 cell viability due to the application of doxorubicin (5 or 10 μM), cyclophosphamide (25 μM) or fluorouracil (25 or 50 μM), compounds **5–7** and their compositions

Drug	Drug dose	Drug action, %	Compound tested (200 μM)	Joint action, %	Σ	Observed effect
-	0 μM	–	**5**	24.3	–	–
			**6**	14.8		
			**7**	36.3		
Doxorubicin	5 μM	72.1	**5**	79.3	96.4	Ant (~17%)
			**6**	72.9	86.9	Ant (~14%)
			**7**	82.9	108.4	Ant (~26%)
	10 μM	70.1	**5**	74.1	94.4	Ant (~20%)
			**6**	68.1	84.9	Ant (~17%)
			**7**	78.5	106.4	Ant (~28%)
Cyclophosphamide	25 μM	11.8	**5**	43.8	36.1	Syn (~8%)
			**6**	41.1	26.6	Syn (~15%)
			**7**	41.8	48.1	Ant (~6%)
Fluorouracil	25 μM	59.9	**5**	65.8	84.2	Ant (~18%)
			**6**	64.3	74.7	Ant (~10%)
			**7**	66.8	96.2	Ant (~29%)
	50 μM	54.0	**5**	60.8	78.3	Ant (~18%)
			**6**	62.1	68.8	Ant (~7%)
			**7**	62.9	90.3	Ant (~27%)

Σ – summarized effect of the drug and compound itself; Ant – antagonizm; Syn – synergism.
